# Large language model trained on clinical oncology data predicts cancer progression

**DOI:** 10.1038/s41746-025-01780-2

**Published:** 2025-07-02

**Authors:** Menglei Zhu, Hui Lin, Jue Jiang, Abbas J. Jinia, Justin Jee, Karl Pichotta, Michele Waters, Doori Rose, Nikolaus Schultz, Sulov Chalise, Lohit Valleru, Olivier Morin, Jean Moran, Joseph O. Deasy, Shirin Pilai, Chelsea Nichols, Gregory Riely, Lior Z. Braunstein, Anyi Li

**Affiliations:** 1 Memorial Sloan Kettering Cancer Center, New York, NY USA; 2 University of California San Francisco, San Francisco, CA USA

**Keywords:** Cancer models

## Abstract

Subspecialty knowledge barriers have limited the adoption of large language models (LLMs) in oncology. We introduce Woollie, an open-source, oncology-specific LLM trained on real-world data from Memorial Sloan Kettering Cancer Center (MSK) across lung, breast, prostate, pancreatic, and colorectal cancers, with external validation using University of California, San Francisco (UCSF) data. Woollie surpasses ChatGPT in medical benchmarks and excels in eight non-medical benchmarks. Analyzing 39,319 radiology impression notes from 4002 patients, it achieved an overall area under the receiver operating characteristic curve (AUROC) of 0.97 for cancer progression prediction on MSK data, including a notable 0.98 AUROC for pancreatic cancer. On UCSF data, it achieved an overall AUROC of 0.88, excelling in lung cancer detection with an AUROC of 0.95. As the first oncology specific LLM validated across institutions, Woollie demonstrates high accuracy and consistency across cancer types, underscoring its potential to enhance cancer progression analysis.

## Introduction

The advent of ChatGPT has sparked widespread excitement about the potential for Artificial Intelligence (AI) to augment various domains of human endeavor^1–3^. Initial explorations of large language models (LLMs) such as ChatGPT in healthcare^4,5^ have yielded promising results across numerous medical fields^6–8^. Oncology, in particular, relies heavily on the nuanced understanding of clinical histories and disease progression. The effectiveness of treatments is closely linked to how cancer responds, as observed through radiological or pathological assessments. These critical data points are often stored as real-world data (RWD) in non-standardized and unstructured formats, making them challenging to access and interpret. The capability of LLMs to decipher and analyze such complex, unstructured data holds transformative potential for improving cancer care, benefiting both clinicians, researchers, and patients.

Privacy concerns have hampered the adoption of closed-source LLMs in clinical settings, compounded by studies indicating notable performance deficiencies in these models within healthcare environments^9^. Moreover, the integration of general domain LLMs into healthcare is challenged by the necessity for domain-specific knowledge, fine-tuning with proprietary clinical data, and the absence of evaluations by domain experts. Conversely, the rise of open-source versions of LLM, inspired by recent advancements like Google’s FLAN-T5^10^, META’s Llama^11^, and Llama 2^12^, has ignited interest across various fields. These open-source models mitigate privacy concerns but face hurdles in adapting and fine-tuning for oncology due to the limited specialized domain background and knowledge from the open-source community. Consequently, the feasibility of applying open-source LLMs directly in clinical oncology practice remains uncertain, indicating a need for further exploration and development to bridge these gaps.

In the medical domain, numerous studies have demonstrated the improved performance of domain-specific foundation LLMs, including BioMedLM^13^, BioGPT^14^, GatorTron^15^, MedPaLM^16^, MedPalM2^17^, and BioMistral^18^. These high performance claims are typically based on standard medical benchmarks such as PubMedQA^19^ and USMLE^6^, whose training datasets are often available during the model’s pre-training phase. However, achieving high scores on these benchmarks does not necessarily equate to increased effectiveness when using RWD in healthcare practices, especially in the oncology sector. Like their general domain counterparts, these specialized medical models frequently lack in-depth expertise in oncology and have not been validated across multiple institutions. This absence of cross-institutional validation restricts their practical use and trust in oncological applications.

Addressing patient privacy concerns, the need for specialized oncology knowledge, the complexity of using real-world data, and the requirement of cross-institutional validation, we created Woollie—a dedicated LLM for oncological radiology reports, based on the open-source Llama (Llama 1)^11^ models from META. We extensively evaluated Woollie in various configurations, from a smaller 7 billion (B) parameter model to larger, more resource-intensive 13B, 33B, and 65B parameter models. Our strategy involved a layered approach to integrating broad oncology knowledge by refining Woollie’s analytical skills through a stacked alignment process. This in-depth investigation included the alignment and evaluation of fourteen unique Woollie models against established benchmarks, such as medical domain tests, including PubMedQA^19^, MedMCQA^20^, and USMLE^17^. We also assessed how different types of prompts affected the model’s tuning. Furthermore, we delved into scaling laws to analyze how model size impacts its effectiveness, providing critical insights into how various Woollie models perform within the oncology domain.

An essential contribution of this work lies in leveraging an advanced, large-scale LLM to analyze real-world clinical oncology data. Our study utilized a specific RWD from Memorial Sloan Kettering Cancer Center (MSK), enabling us to test Woollie’s proficiency in oncology-related tasks rigorously. By tapping into MSK’s extensive oncologic domain knowledge, we thoroughly evaluated Woollie’s ability to interpret radiology reports, examining 38,719 radiology impressions curated by radiologists from 3402 patients across five types of cancer: lung, breast, pancreatic, prostate, and colorectal. For oncologists, the impression section of radiology reports provides comprehensive insights into tumor location, size, and changes over time—critical factors for determining cancer staging and guiding treatment strategies. Tracking tumor progression through radiology is essential for assessing the effectiveness of treatments and guiding further clinical management. Leveraging radiology impression notes, we assessed Woollie’s effectiveness in monitoring cancer progression and extracting pivotal information of cancer biology, identifying patterns of metastatic spread across a vast array of medical information.

A crucial element of this work was demonstrating the model’s cross-institutional generalizability and applicability, highlighting its relevance in various healthcare contexts. We extended our validation to include an independent dataset of 600 radiology impressions involving 600 unique patients from the University of California, San Francisco (UCSF), focusing on lung, breast, and prostate cancers—different from those in the MSK dataset. This step was essential to confirm Woollie’s ability to consistently apply its oncology expertise across various settings, establishing its broad utility and effectiveness in diverse healthcare environments.

## Results

### Performance assessment of Woollie models in standard non-medical benchmarks with improved outcomes in medical domains

**Fig. 1:**
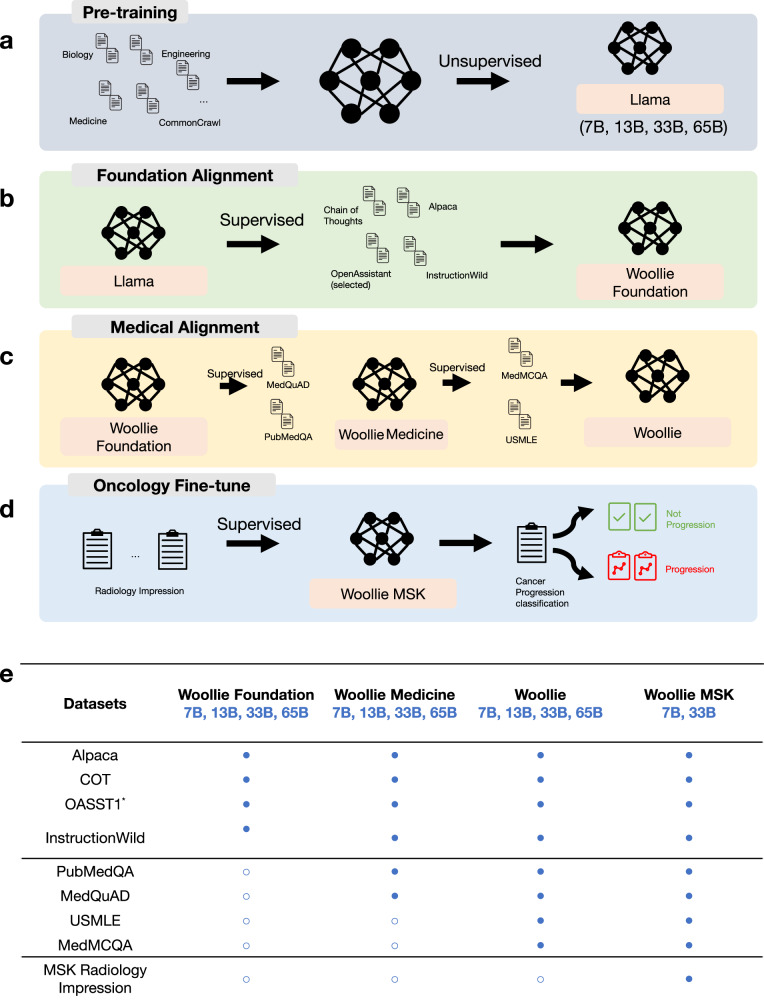
Stacked refinement of Woollie models, from pre-training to oncology domain fine-tuning. **a** Overview of the pre-training process for the baseline Llama models, highlighting unsupervised training with a dataset featuring 1.4 trillion tokens, including the Common Crawl dataset and knowledge from medicine, engineering, mathematics, biology, etc. This stage encodes foundational knowledge across various domains into the models, available in four sizes: 7 billion (B), 13 billion, 33 billion, and 65 billion parameters. **b** Description of the domain knowledge alignment process for the baseline Llama models using the Chain of Thoughts (COT), Alpaca, OpenAssistant, and InstructionWild datasets, creating the Woollie Foundation models. This supervised learning step involves training models to answer questions correctly, enhancing their reasoning, logic, and conversational abilities. **c** Further alignment with the general medical and oncology domain using datasets of MedQuAD, PubMedQA, MedMCQA, and USMLE, leading to the development of Woollie Medicine and Woollie. Woollie Medicine is an intermediate model for evaluating the effectiveness of stacked alignment methods. **d** Final fine-tuning within the oncology domain using a proprietary MSK dataset of 38,719 radiology reports manually curated by radiologists from 3402 patients across five cancer types: breast, colorectal, lung, pancreatic, and prostate. This step trains the Woollie MSK models (7B and 33B versions) to determine tumor progression, with models being benchmarked against a test dataset and cross-institutionally validated with a UCSF dataset covering lung, breast, and prostate cancers. **e** The summary of fourteen Woollie models detailing the datasets used for alignment and fine-tuning. The table categorizes the datasets into three groups: reasoning, logic, and conversation; general medical domain; and oncology domain, including the proprietary MSK dataset of radiology impressions. asterisk notes the selective use of 10,000 high-quality examples from the OpenAssistant dataset (OASST1), which contains 160,000 human-created and annotated conversations in various languages.

**Fig. 2:**
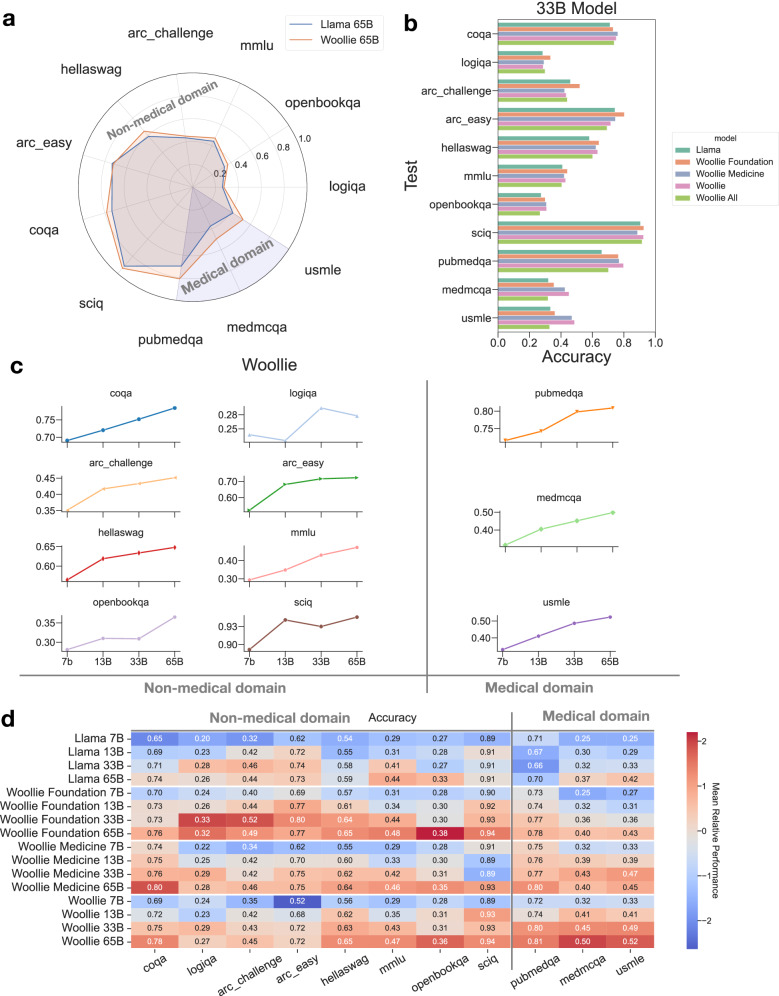
Performance comparisons of Woollie models on benchmarks, the influence of model size, and improvements in the medical domain. **a** The stacked alignment strategy mitigates catastrophic forgetting in LLM. When comparing the baseline Llama 65B model to the Woollie 65B model, modest improvements are observed in standard benchmarks testing reasoning and logic in non-medical domains. In the medical domain, however, significant improvements are evident. The performance in the medical domain is accentuated in shaded sections, illustrating how stacked alignment enhances performance while preserving capabilities in general domains. **b** Comparison of all stacked aligned 33B models (Woollie Foundation, Woollie Medicine, and Woollie) with non-stacked aligned 33B models (Woollie All). The results clearly demonstrate that stacked alignment significantly improves model performance incrementally, whereas non-stacked alignment leads to catastrophic forgetting, resulting in poorer performance. **c** A scaling study plotted the performance of Woollie models against model sizes ranging from 7B to 65B parameters across 11 tests. The results suggest that larger models generally achieve better performance, though there is a noticeable plateau in performance enhancement between the 33B and 65B models. This informs the decision-making process for model selection in clinical applications, considering the balance between performance and resource consumption. **d** A detailed performance comparison among twelve Woollie and four Llama models across 11 tests is depicted in a heatmap. The color intensity in each cell reflects the mean relative performance in each test. The heatmap is divided into non-medical domains on the left and medical domains on the right, categorizing the models into Llama, Woollie Foundation, Woollie Medicine, and Woollie. This visualization underscores the performance improvements achieved through stacked alignment, with a clear transition from left to right, highlighting advancements in medical and oncology domains across the models.

We trained a family of fourteen distinct Woollie models using a stacked alignment and fine-tuning process. This process began with pretrained open-source Llama models (Fig. 1a) and progressively built upon each iteration using a variety of datasets (Fig. 1b, c). The performance of Woollie models and baseline Llama models was assessed across 11 standard benchmarks in four different sizes—7B, 13B, 33B, and 65B parameters, resulting in a total of 176 tests. We assessed the models’ performance by calculating accuracy, precision, F1 scores, and Matthews Correlation Coefficients (MCC) on the test datasets. For standard benchmarks involving multiple classes, we computed the macro-average of all metrics except accuracy (Supplementary Table 1). Accuracy was chosen as the primary metric for comparison, as most benchmarks feature balanced classes with equal importance, and the primary focus is on the model’s ability to predict the correct classes. Additionally, accuracy is widely used in open leaderboards for open-source LLMs, making it easier for performance comparisons. Other metrics show consistent trends, aligning with the overall model performance. When compared to Llama models, Woollie models exhibited superior capabilities in logic, conversation, and reasoning tasks. In non-medical benchmarks such as OpenbookQA^21^, LogiQA^22^, and MMLU^23^, particularly in conversation-centric tests like COQA^24^ and HellaSWAG^25^, the larger Woollie models (13B, 33B, and 65B) outperformed their Llama equivalents, with the 65B model showing the most significant advantage (Fig. 2a, Supplementary Fig. 1a). For the COQA benchmark, the Woollie 65B model achieved an accuracy of 0.78 compared to 0.74 for the Llama 65B model, and an F1 score of 0.82 versus 0.77.

Catastrophic forgetting^26^, a common issue where an LLM loses previously learned knowledge during fine-tuning, is mitigated in Woollie models through the stacked alignment strategy. This approach allows us to observe performance improvements by progressively building on previously trained models. A comparison among Llama, Woollie Foundation, Woollie Medicine, and Woollie models clearly demonstrates incremental performance improvements across various benchmarks (Fig. 2b, Supplementary Fig. 1b).

To better visualize the impact of stacked alignment, we also compared models trained by simply concatenating all training datasets, labeled as Woollie All. While Woollie All outperformed the baseline Llama models, significant performance gaps were evident when compared to Woollie models aligned with the stacked alignment strategy. These gaps were particularly pronounced in non-medical benchmarks and even more so in medical benchmarks.

For instance, in the 33B model evaluations, Woollie 33B achieved an accuracy of 0.43 on MMLU, compared to 0.40 for Woollie All 33B. Similarly, on PubMedQA, Woollie 33B achieved an accuracy of 0.79, significantly outperforming Woollie All 33B at 0.70. These results suggest that the stacked alignment strategy effectively counteracts the problem of catastrophic forgetting. As the comparison between 7B, 13B, and 33B models clearly demonstrated the performance trends, we decided to conclude training at Woollie All 33B.

In the medical domain, the Woollie models significantly outperformed the Llama models in tests such as PubMedQA, MedMCQA, and USMLE. Specifically, the Woollie 65B model marked substantial improvements, achieving accuracies of 0.81 on PubMedQA, 0.50 on MedMCQA, and 0.52 on USMLE—exceeding the Llama 65B model’s scores of 0.70, 0.37, and 0.42, respectively (Fig. 2a). Notably, the Woollie 65B model’s performance on PubMedQA matched GPT-4’s accuracy of 0.804 and significantly surpassed ChatGPT (3.5-turbo)’s accuracy of 0.716^5^, despite being considerably smaller than the GPT-3 (175B parameters)^27^ and GPT-4^28^, which is estimated to have over 1 trillion parameters. These results underscore Woollie’s efficacy in general medical contexts and validate the effectiveness of our stacked alignment strategy, which adeptly incorporates domain-specific knowledge while maintaining robust performance across broader conversational and reasoning tasks.

### Scaling Woollie models and comprehensive comparisons across model variants

We performed a scaling study to evaluate the impact of size, comparing performance across models ranging from 7B to 65B parameters. The study included Llama, Woollie Foundation, Woollie Medicine, and Woollie models. Llama showed that performance generally improved with size, except on the PubMedQA tests, where the accuracy was 0.71 for 7B, 0.67 for 13B, 0.66 for 33B, and 0.70 for 65B (Supplementary Fig. 1c). After alignments, all models confirmed the expected scaling benefits. For example, in the Woollie model, the accuracy on PubMedQA improved with size: 0.72 for 7B, 0.74 for 13B, 0.80 for 33B, and 0.81 for 65B (Fig. 2c).

This trend affirmed that models with a more extensive parameter base before alignment yielded better outcomes, with the performance scaling almost linearly with sizes between 7B and 33B. The trend suggested that the alignment’s effectiveness is contingent upon the quality of the baseline model’s pre-training—the more robust the baseline, the better the performance of the aligned models. Nevertheless, a modest performance improvement was noted in this scaling trend when comparing the 33B and 65B models, both pre-trained on datasets of identical size (1.4 trillion tokens). For instance, on the PubMedQA benchmark, the Woollie 33B model achieved an accuracy of 0.7980, while the 65B model reached 0.8092. This observation indicates that beyond a certain point, the scaling law in aligned models may be more influenced by data quality than quantity alone.

Our assessments concluded with a detailed performance evaluation of Llama, Woollie Foundation, Woollie Medicine, and Woollie models, visually presented through a heatmap of accuracies for precise comparative analysis. This heatmap used varying shades of color to indicate performance levels across different test categories, clearly separating results from non-medical and medical domains (Fig. 2d). As more medical datasets were integrated into the alignment process, a distinct trend appeared: non-medical performance slightly declined while medical domain proficiency increased. This specialization trade-off is depicted in the heatmap through a gradient change, where color intensity fades in non-medical domains and deepens in medical domains, illustrating the models’ shifting focus and capabilities. Despite the marginal decrease in non-medical domain performance among the aligned models, their capabilities remained robust and competitive. Our approach fortified the model’s foundation and optimized it for oncology-specific applications, demonstrating improved performance upon further fine-tuning with oncology datasets.

### Evaluation of Woollie models performance on oncologic radiology datasets

**Fig. 3:**
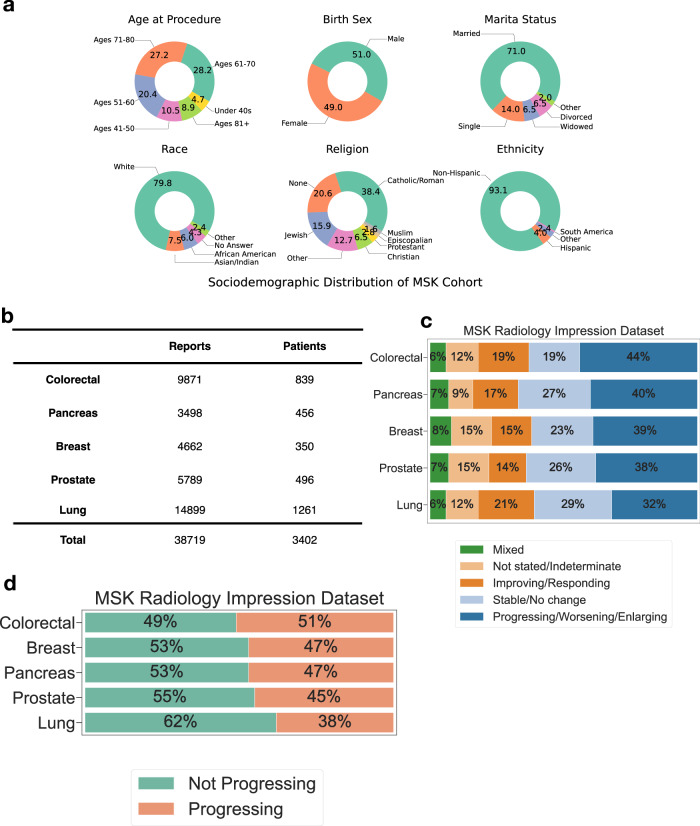
Sociodemographic characteristics and patient cohort distribution in the MSK radiology impression dataset. **a** Sociodemographic distribution of the MSK radiology impression dataset, categorized by “Age at Procedure,” “Birth Sex,” “Marital Status,” “Race,” “Religion,” and “Ethnicity.” **b** Tables provide the number of reports and unique patients for each cancer type. **c** The MSK radiology impression dataset, manually curated by radiologists, classifies cancer progression into five categories: Progressing/Worsening/Enlarging, Stable/No change, Improving/Responding, Not Stated/Indeterminate, and Mixed. For each cancer type—colorectal, pancreatic, breast, prostate, lung—we detail the number of reports and the distribution percentage of these five labels within each type. **d** Distribution of the MSK radiology impression dataset, featuring labels for progression and non-progression across five cancers: breast, colorectal, lung, prostate, and pancreatic, comprising 38,719 reports from 3402 patients.

Leveraging Woollie’s strong performance in medical benchmarks, we aim to support oncologists by monitoring cancer progression and understanding disease pathways. We fine-tuned and tested Woollie’s capability using real-world data of 38,719 radiology impressions from 3401 patients with five different cancers: breast, colorectal, lung, pancreatic, and prostate. We analyzed and plotted the sociodemographic distribution of the patient cohort included in this dataset (Fig. 3a), and listed number of radiology impression notes and the associated number of patients in each cancer (Fig. 3b). The sociodemographic distribution plot categorizes the data by “Age at Procedure,” “Birth Sex,” “Marital Status,” “Race,” “Religion,” and “Ethnicity.” Notably, over 95% of the cohort is aged 40 or older. Approximately 80% of the patients identify as White, which is slightly higher than the national average of 75% as of 2024. In contrast, 6% of the cohort identify as African American, below the national average of 13%. The representation of Asian/Indian individuals is 7.5%, slightly above the national average^29^. These radiology impressions, gathered from MSK^30^, were thoroughly reviewed and labeled by radiologists with five categories indicating cancer progression: “Progressing/Worsening/Enlarging,” “Stable/No change,” “Improving/Responding,” “Not stated/Indeterminate,” and “Mixed” (Fig. 3c). We simplified the classification into a binary system, grouping “Mixed” and “Progressing/Worsening/Enlarging” as positive indicators labeled “Progressing,” while all other cases were classified as negative indicators labeled “Not Progressing.” The proportion of “Not Progressing” labels is roughly equal to that of “Progressing” labels, except in lung cancer cases, which show a slight skew toward “Not Progressing” (Fig. 3d).

**Fig. 4:**
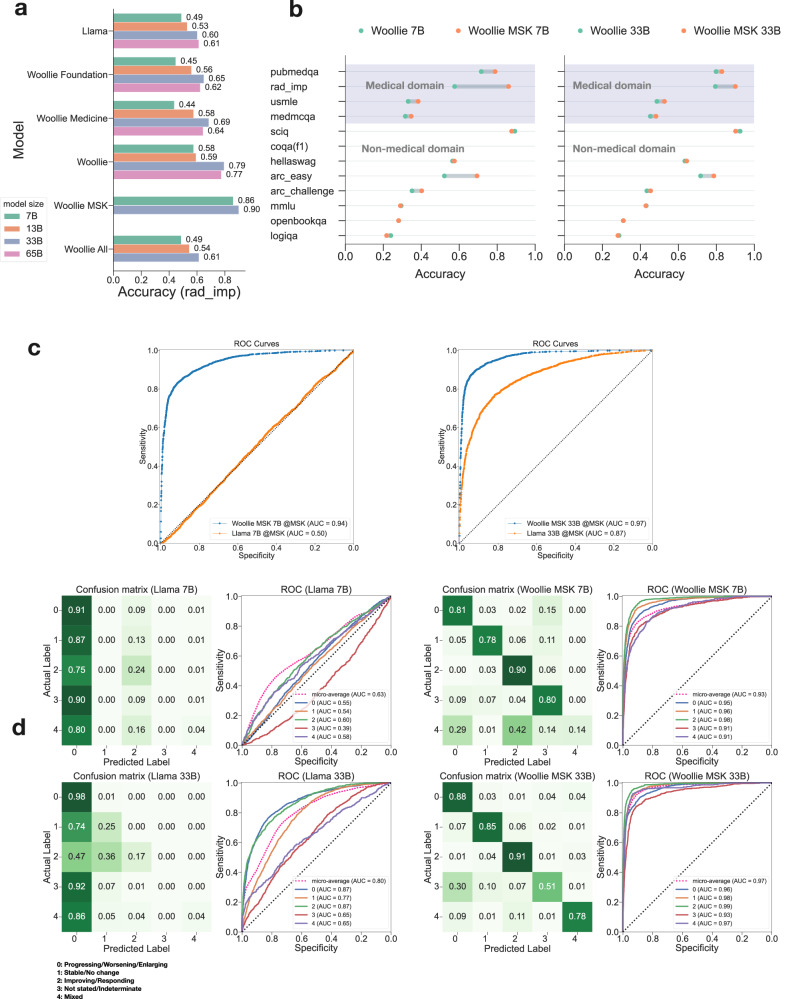
High-performance cancer progression prediction by Woollie MSK models fine-tuned on MSK oncology data, including their performance metrics and comparisons with other models. **a** Comparison of Llama, Woollie Foundation, Woollie Medicine, Woollie, Woollie MSK models, and non-stacked aligned Woollie All models on the MSK radiology impression dataset (rad_imp) for binary classification of cancer progression. The Woollie 33B model achieves an accuracy of 0.79, outperforming the 65B model at 0.77. Fine-tuned Woollie MSK models achieve superior accuracies of 0.86 (7B) and 0.90 (33B) across all five cancer types. Non-stacked aligned Woollie All models lag behind the stacked aligned Woollie models. **b** Woollie MSK models, fine-tuned on top of existing Woollie models, show improvement in the general medical domain on tests like PubMedQA, USMLE, and MedMCQA. Fine-tuning on the MSK oncology dataset enhances performance: Woollie MSK 33B’s accuracy increased to 0.83 from 0.80 in PubMedQA, 0.48 from 0.45 in MedMCQA, and 0.53 from 0.49 in USMLE. The Woollie MSK 7B model similarly shows gains, with accuracies improving notably across all tests. **c** ROC plot illustrating the performance of Woollie MSK 7B on the MSK dataset, with a significant increase from a 0.50 AUROC for the baseline Llama model to 0.94 for Woollie MSK 7B. The right panel shows the larger Woollie MSK 33B model reaching an AUROC of 0.97, compared to 0.87 for the Llama model. **d** Comparative performance analysis between the Llama 7B and Woollie MSK 7B models five labels reveals a significantly higher (*p* < 0.001) micro-average AUROC for Woollie MSK 7B at 0.93 on the right, compared to 0.63 for Llama 7B on the left. A comparison between Llama 33B and Woollie MSK 33B models on the same dataset and labels shows an AUROC of 0.97 for Woollie MSK 33B versus 0.8 for Llama 33B. Furthermore, the Woollie MSK 33B model demonstrates enhanced performance in the confusion matrix with a higher accuracy of 0.82 compared to 0.76 for Woollie MSK 7B.

To gauge the extent of knowledge transfer during fine-tuning, we also compared the performance of non-finetuned Woollie Foundation, Woollie Medicine, Woollie All, Woollie models, as well as baseline Llama models, on this MSK radiology impression dataset, denoted as “rad_imp” (Fig. 4a). The performance comparison between the “stacked aligned” Woollie models, “not stacked aligned” Woollie All models, and baseline Llama models provides further evidence of the significant impact of the alignment strategy on performance, particularly on proprietary datasets that were not exposed during pretraining. The Woollie All models (7B, 13B, and 33B) demonstrate higher accuracy compared to their corresponding Llama models. However, their performance remains significantly lower than that of the Woollie models. The binary classification results indicated that larger models generally yield better performance; however, interestingly, the Woollie 65B model’s accuracy slightly lagged behind the 33B model, with accuracy of 0.77 versus 0.79 for the 33B model.

Considering our observations on the scaling effects, we focused our model selection for further oncologic fine-tuning on the Woollie 7B and 33B models only. This decision was reinforced by comparing the performance of the 65B and 33B models on the “rad_imp” test and by noting that the performance decrease of the 65B model over the 33B model (Fig. 4a). In addition, when evaluating standard medical benchmarks such as PubMedQA, MedMCQA, and USMLE, the overall accuracy difference is minimal, with the 33B model scoring 0.589 and the 65B model scoring 0.592. Including the 7B model in our analysis serves a strategic purpose, allowing us to explore the impact of model size scaling and evaluate the feasibility of deploying smaller models in clinical settings. The 7B model’s smaller parameters makes it an attractive option for widespread clinical use, capable of running on a single GPU for inference, thus eliminating the need for specialized centralized computing resources. We evaluated the memory footprint, inference time, and energy consumption of the 7B and 33B models (Supplementary Table 2), with detailed findings discussed in the Discussion section.

### Predictive analysis of cancer progression using real-world oncologic aligned Woollie models

Building upon the Woollie models, we enhanced their capabilities by fine-tuning them with the MSK radiology impression dataset. This refinement led to the development of two specialized models: Woollie MSK 7B and Woollie MSK 33B, both of which exhibited substantial improved performance on this dataset. When evaluating cancer progression as a binary classification within the test dataset, Woollie MSK 7B outperformed all competing 65B models (Llama, Woolie Foundation, Woollie Medicine, Woollie), achieving an overall accuracy of 0.86 across all disease sites in the “rad_imp” test. Woollie MSK 33B showed even greater efficacy, achieving an overall accuracy of 0.90 (Fig. 4a).

Interestingly, the models fine-tuned with the MSK dataset also significantly improved general medical domain benchmarks. Compared to the baseline Woollie models, the accuracy of Woollie MSK 33B increased from 0.80 to 0.83 in PubMedQA, from 0.45 to 0.48 in MedMCQA, and from 0.49 to 0.53 in USMLE. A similar trend was observed in the smaller Woollie MSK 7B model, where accuracy improved from 0.72 to 0.79 in PubMedQA, from 0.32 to 0.35 in MedMCQA, and from 0.33 to 0.38 in USMLE (Fig. 4b). These improvements were statistically significant (*p* < 0.001). These enhancements highlight Woollie’s capacity to integrate and apply medical knowledge effectively, emphasizing its adaptability and reinforcing its role as a versatile foundational model. This also illustrates that data quality is critical in enhancing model performance across various disease sites rather than merely the quantity of data.

We examined the receiver operating characteristic (ROC) curve to further analyze cancer progression through model sensitivity and specificity. As Woollie is a generative model, we calculated the ROC using the logits of the generated tokens in its predictions (Supplementary Note 2). In addition to using accuracy as a performance indicator, we also calculated the Area Under the Receiver Operating Characteristic Curve (AUROC). AUROC assesses the model’s performance independently of specific classification thresholds, making it particularly valuable in scenarios where thresholds may vary. Furthermore, it serves as a standard baseline for comparing different models and training approaches, ensuring consistency across experiments. Comparing the Woollie MSK models to the Llama models, Woollie MSK 7B achieved an impressive AUROC of 0.94 across all disease sites, significantly outperforming the base Llama 7B model’s AUROC of 0.50. Similarly, Woollie MSK 33B surpassed the baseline Llama 33B model with an AUROC of 0.97, compared to 0.87 (Fig. 4c). These models were particularly effective in detecting pancreatic cancer, achieving an AUROC of 0.98 and an accuracy of 0.92 (Supplementary Fig. 2a, b). The details of the metric selection for cancer progression prediction are provided in the Methods section.

Beyond binary classification, we noticed notable enhancements in performance when evaluating the models’ capacity to differentiate between the original five labels from the MSK dataset. The micro-averaged AUROC for the 7B models saw a significant increase from 0.63 to 0.93 comparing to Llama. For the 33B models, it escalated from 0.80 to 0.97 (Fig. 4d). This substantial improvement highlights the models’ refined ability to discern complex clinical labels, which is vital for practical applications in oncology. These scores provide a precise quantitative evaluation of the model’s accuracy and capability to accurately classify clinical conditions and critical factors in clinical environments.

### Woollie shows exceptional generality via cross-institutional validation

**Fig. 5:**
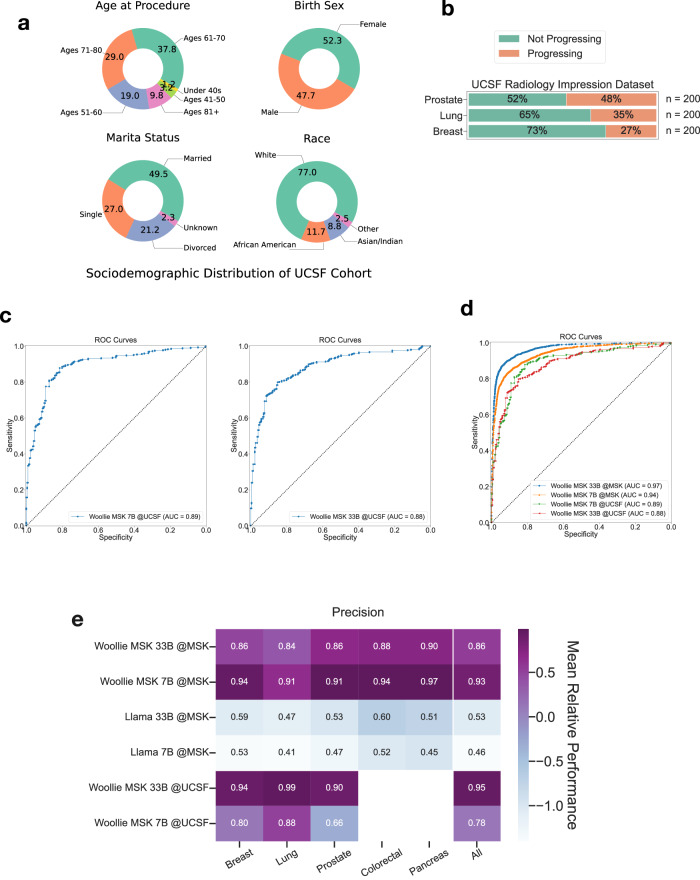
Cross-institution validation of model performance in predicting cancer progression on MSK and UCSF datasets. **a** Sociodemographic distribution of the UCSF radiology impression dataset, used exclusively as an independent validation dataset. This dataset was not used for Woollie MSK fine-tuning. The UCSF dataset has different sociodemographic distribution than MSK dataset. **b** UCSF dataset includes 600 reports from 600 unique patients, covering prostate, lung, and breast cancers, distinct from the MSK data. **c** Fine-tuned with MSK oncology data, ROC curves for Woollie MSK 7B and 33B models demonstrate performance on the UCSF dataset. The Woollie MSK 7B model achieves an AUROC of 0.89, slightly better than the 0.88 for the Woollie MSK 33B, suggesting that smaller models may outperform larger ones in this dataset due to less bias but increased variance. **d** Comparison of Woollie MSK models on both MSK and UCSF datasets shows superior performance on MSK data, though the knowledge transfer to UCSF is clear. Despite lagging behind the MSK performance, the trend is consistent, indicating effective cross-institutional validation in cancer progression on an open-source LLM. **e** Precision scores are visualized on a heatmap, with varying color intensities indicating effectiveness in detecting different types of cancer, noting the absence of data for colorectal and pancreatic cancers in the UCSF dataset. Precision scores are crucial for closely monitoring progressive cases. While Woollie MSK 7B shows higher AUROC scores on the UCSF dataset, Woollie MSK 33B excels in precision, significantly notably with a score of 0.99 in detecting lung cancer. The fine-tuned Woollie models significantly outperform Llama models in accurately tracking cancer progression, underscoring their practical application of inter-institutionally transferred knowledge.

To further assess Woollie’s capability to apply its knowledge of cancer progression beyond merely replicating learned language patterns, we utilized an independent dataset from UCSF. This dataset comprised 600 radiology impressions covering breast, lung, and prostate cancers, with 200 impressions per cancer type from various patients. The UCSF dataset has a different sociodemographic distribution compared to the MSK dataset (Fig. 5a). Each impression was manually labeled as either “Progressing” or “Not Progressing” (Fig. 5b). The Woollie MSK 7B and 33B models were tested on this dataset and achieved AUROCs of 0.89 and 0.88, with accuracies of 0.80 and 0.78, respectively, in detecting cancer progression (Fig. 5c). These results are significant as the models successfully navigated unfamiliar formats and terminologies do not present in the MSK dataset. This success underscores that specialized training enhances performance within a given dataset and equips the model to generalize to other datasets with varying characteristics. Notably, in detecting lung cancer progression, the models achieved an AUROC of 0.95 and an accuracy of 0.88 (Supplementary Fig. 3). Although there was a slight drop in performance compared to the MSK data, the Woollie models demonstrated effective knowledge transfer, affirming their utility in diverse oncological settings across institutions.

ROC curves for all models (Fig. 5d) highlighted the superior performance of the Woollie MSK models. While the Woollie MSK 33B model outperformed the 7B model on the MSK dataset, it showed a slight decrease in AUROC on the UCSF dataset (0.88 compared to 0.89 for the 7B model). This discrepancy suggests a common trade-off in larger models like the 33B, which typically exhibit low bias but high variance, making them adept at fitting training data closely. This trade-off highlights the need for careful model selection and evaluation, considering factors such as model size, computational resources, and the specific requirements of clinical settings. In addition to using AUROC as a metric, this study was driven by the potential use case of deploying LLMs as real-time assistants for oncologists while they enter patient information into electronic health record systems. For such clinical applications, the precision of the model’s predictions is critical, as it ensures oncologists can confidently asking the model to identify cancer progression precisely. A precision comparison across models, including the baseline Llama (Fig. 5e), using both MSK and UCSF datasets revealed that although the 33B model’s AUROC was slightly lower than the 7B’s, its precision in detecting cancer progression was higher at 0.95 compared to 0.78, with high precision in detecting lung cancer progression at 0.99.

### Woollie distilled the primary topics and disease trajectories from the report

**Fig. 6:**
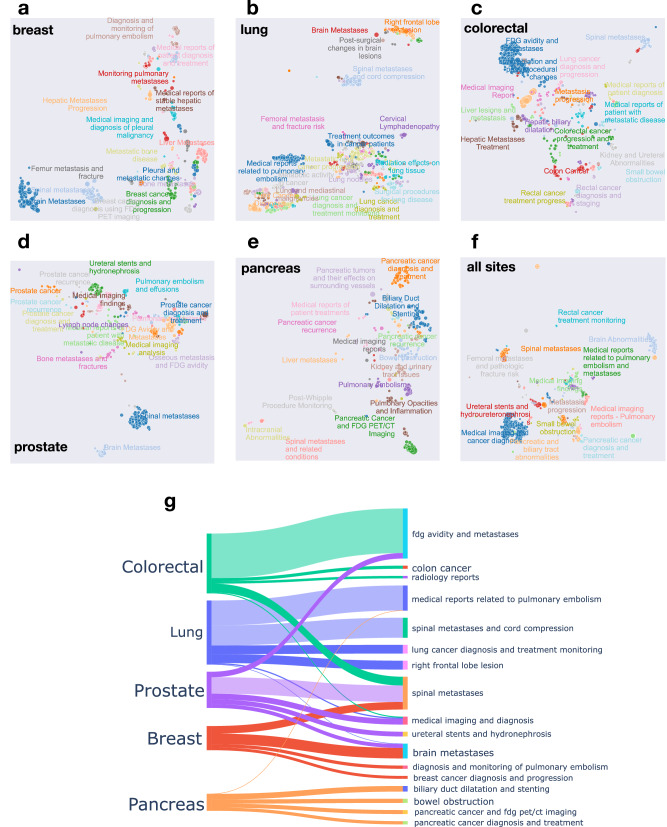
Model parsing of disease trajectories and biology among different malignancies. Salient topics from radiology reports using Woollie MSK 33B on reports from MSK patients with cancers of the **a** breast, **b** lung, **c** colorectal, **d** prostate, and **e** pancreatic. **f** Summarization of the salient topics among all diseases, notably enriched for sites of distant metastatic seeing. **g** Sankey plots demonstrating trajectories of metastatic disease across five cancer types.

Despite the complexity often associated with large language models, our study aimed to assess whether Woollie could identify and synthesize key themes and disease trajectories from medical records. We posited that LLM-based topic modeling would outperform conventional methods like Latent Dirichlet Allocation (LDA)^31^ due to the model’s capacity to navigate complex linguistic features, idiomatic expressions, and contextual nuances typical of clinical narratives. Such advanced topic modeling proves especially useful in oncology contexts. We applied radiology impression data to extract insights regarding the nature of tumor progression. Woollie distilled the primary themes of each report (Fig. 6a–f), exhibiting not just the presence or absence of disease progression but further detailing the sites of disease progression. The model deciphered the rationale behind each imaging study in this analysis—diagnosing a specific condition like a pulmonary embolism or monitoring metastatic disease. Notably, Woollie plausibly identified the most frequent sites of metastatic involvement that are otherwise known to be associated with the evaluated cancers (e.g., brain or bone metastases among the cohort with breast cancer or hepatic metastases for those with colorectal cancer^32,33^).

Woollie’s ability to extract global features like topics related to cancer biology is unsurprising given its pretraining, alignment, and fine-tuning processes. Additionally, we evaluated the model’s understanding of the local features using local interpretable model-agnostic explanations (LIME)^34^, rather than treating the model as a black box. The results demonstrate that the model has effectively learned and utilizes local features to inform its classifications (Supplementary Figs. 4, 5, Supplementary Note 3). Woollie highlights terms like “FDG” and “Increase” in notes labeled as “Progressing,” while also recognizing the influence of words such as “small” and “mild” in notes classified as “Not Progressing.” These local features, which play a key role in identifying cancer progression, demonstrate the model’s understanding of cancer biology and align with human interpretation. Given the Woollie’s capability of topic modeling and local feature extraction, it provides confidence in the model’s explainability and its potential for clinical applications.

The Sankey plot (Fig. 6g) visually represents the disease trajectory across different cancer sites within patients from diagnosis onward. This plot offers insightful visual analytics on how patients’ cancer types may evolve or spread to other sites over time, highlighting potential metastasis pathways or indicating typical trajectories in disease progression. By tracing these trajectories, we can gain a deeper understanding of cancer biology, including patterns of metastasis, the evolution of cancerous lesions over time, and how different types of cancer might share common pathways or diverge into distinct evolutionary routes. Our approach, using a single model, contrasts with similar studies on metastatic disease that employ multiple models^35^, offering potential for new insights into disease progression pathways that have not been thoroughly explored yet.

## Discussion

The findings of this study have several important implications for the integration of LLMs into the field of oncology. (1) It is possible to align and fine-tune LLMs within the oncology domain without losing prior knowledge. This can be achieved by selecting high-quality datasets and employing a stacked alignment methodology. By preserving general domain competencies of reasoning, conversation, and information extraction while enhancing medical domain proficiency, the aligned models demonstrated a strong foundation for further specialization. This approach has shown the ability in preventing catastrophic forgetting, ensuring the model retains comprehensive capabilities while specializing in medical knowledge. Additionally, choosing a baseline model with adequate parameters is crucial for successful alignment and fine-tuning, as the knowledge acquired during pre-training plays a significant role in performance on downstream tasks. Our scaling studies indicate that larger models significantly outperform smaller ones. (2) Baseline benchmarks on LLMs should not be the sole metrics for evaluating model performance. Our results highlight the importance of cross-institutional validation. While some performance degradation is expected when encountering new datasets, the overall performance remains remarkable. The model’s generalizability is validated, with performance metrics of AUROC illustrating the model’s capability to classify tumor progression accurately. (3) Knowledge transfer between proprietary data and standard benchmarks is not evident. For example, the Guanaco 65B model^36^, an enhancement of the Llama 65B model through fine-tuning, attained 99% of ChatGPT’s performance according to the Vicuna benchmark^37^ and was at the forefront of the open-source LLM leaderboard. When we evaluated its performance within our specific field of oncology, despite its substantial 65 billion parameters—a figure nine times greater than that of a 7 billion parameter model—its performance did not measure up to our Woollie MSK 7B model. It even fell short compared to the Woollie 33B model without finetuning on the MSK dataset (Supplementary Fig. 6). (4) The inclusion of oncology domain knowledge from radiology impression has also enhanced the model’s performance on standard medical benchmarks like PubMedQA (Fig. 4b). This underscores the LLM’s role as a foundational model that can adapt to various tasks, particularly in medical contexts. (5) Considering the cost implications, using LLMs in oncology for purposes like tracking cancer progression and analyzing reports proves viable and advantageous. The cross-institution validation of the Woollie model, validated on UCSF data, demonstrates that the smaller 7B model performs comparably and even slightly outperforms its larger 33B counterparts. When comparing the resource usage of deploying 33B models versus smaller 7B models, we found that the 7B model is more economically viable for clinical applications due to its lower memory footprint, reduced energy consumption, and less demanding CPU and GPU requirements. Larger models like the 33B, while powerful, are impacted by their complexity, resulting in slower inference times. Specifically, the 7B model is three times faster than the 33B model (Supplementary Table 2). In clinical workflows, classification requests can arise from various scenarios, such as real-time cancer progression classification while a physician enters data into electronic health records (EHR), or retrospective studies analyzing tumor progression across patient cohorts. 7B model’s efficiency and faster inference times make it significantly more practical and feasible for deployment in a clinical environment as oncologist assistant. This highlights a critical balance between performance and practicality in clinical environments.

The datasets from MSK and UCSF differ in the sociodemographic composition of their patient populations, making it important to evaluate the model’s generalizability across subgroups defined by “Age”, “Birth sex”, and “Race”. To assess this, we computed performance metrics stratified by these variables (Supplementary Table 5a) and conducted statistical comparisons. No significant differences in performance were observed across these demographic groups (*p*-values > 0.1). However, smaller sample sizes within certain age and race subgroups limit our ability to fully exclude the possibility of performance variation. While radiology impression narratives appear largely independent of these demographic factors, underrepresentation in specific subgroups may impact model robustness. Future studies with larger and more balanced cohorts are needed to further clarify these findings. In contrast, model performance differed significantly by cancer type (Supplementary Table 5b), with all associated p-values below 0.01. These variations likely reflect differences in reporting style and diagnostic emphasis across anatomical sites, which influence the content and structure of radiology impressions.

When utilizing Woollie as a classifier, we configured the inference settings with a temperature of 0.01 and top_p of 1.0. This setup effectively minimizes randomness, ensuring deterministic model behavior. Concerns regarding model hallucination were addressed through performance metrics such as AUROC. The high AUROC scores, alignment with oncological knowledge, and localized explainability analyses using LIME strongly suggest that hallucination has been significantly minimized in our use cases. Although hallucination in generative models remains a compelling and critical topic, further research is required to explore its implications in scenarios beyond those examined in this study.

However, it is important to acknowledge that hedging language—such as “possibility,” “cannot exclude,” or “likely”—frequently reflects the inherent uncertainty in radiology impressions. While the use of human-curated labels helps transfer sentiment, contextual understanding, and domain knowledge into the LLM, the specific impact of such uncertain phrasing has not been systematically studied and warrants dedicated investigation, which was beyond the scope of this work. Additionally, reliance solely on free-text radiology impressions may introduce biases tied to institutional reporting styles. Although we illustrate stylistic differences between MSK and UCSF reports (Supplementary Note 6), future work should consider incorporating imaging data into a multimodal LLM framework to mitigate such limitations and enhance model robustness.

Woollie’s capabilities extend beyond mere data interpretation to an in-depth understanding of cancer biology, positioning it well beyond conventional topic modeling methods. Woollie adeptly summarizes the salient themes within medical radiology reports, pinpointing intricate details of disease progression and the exact locations of metastatic spread amidst a wealth of medical data. This proficiency indicates Woollie’s potential not just for comprehending the intent behind medical imaging but also for mapping out and succinctly encapsulating the complex pathways of disease evolution across diverse organ systems (Fig. 6). Such insights are invaluable, as they provide a deeper understanding of the prevalent routes of metastasis associated with various types of cancer, thereby enhancing the scope of oncological assessment and treatment planning.

Our examination of fourteen distinct Woollie models revealed a slight decrease in general performance following domain-specific training that was not otherwise substantial while demonstrating marked improvement in domain-specific knowledge. This contrasts with BERT (Bidirectional Encoder Representations from Transformers) based models, which have been extensively studied in the medical field. The LLM has proven to be an adept multitasking learner. Training tailored to one proprietary dataset improved performance in the oncology domain and enhanced performance across other general medical domains and oncology institutions. Once fine-tuned, an LLM can serve multiple functions across various subspecialties, making it an invaluable tool for oncology clinics and research. Its application extends to knowledge extraction, report generation, and classification, supporting a broad spectrum of medical knowledge requirements.

## Methods

### Hardware and software

Our experimental setup leveraged the advanced resources of MSK’s high-performance computing cluster, utilizing 64 Nvidia A100 GPUs (80GB VRAM) and 30TB of GPFS storage. We employed the Distributed Data Parallel (DDP) framework from PyTorch for model training, adapting our process to utilize varying numbers of GPUs - specifically 4, 8, 16, 32, or 64 GPUs, in alignment with the cluster’s configuration where each node houses 4 GPUs. Resource management and job submission were efficiently handled using IBM Spectrum LSF. The fine-tuning process and subsequent analyses were conducted using Python, ensuring a streamlined and effective workflow.

### Dataset

Our study categorized the training dataset (Fig. 1e) for model alignment and fine-tuning into two primary groups. The non-medical domain aims to enhance the model’s reasoning, question-and-answer, and conversational capabilities to mimic human interaction. This category included: (1) An updated Alpaca dataset (link), augmented with GPT-4-generated content and manually refined to reduce inaccuracies^38,39^. (2) Google’s Chain of Thought (COT) dataset (link) that was modified for instruction-based fine-tuning^40^. (3) An English rendition of the InstructionWild dataset^41^ (link), sourced from Twitter and vetted by human reviewers. (4) A subset of the OpenAssistant dataset (OASST1) (link), containing 160,000 human-created and annotated conversations, from which we selected 10,000 high-quality examples in various languages^42^. The choice of datasets for non-medical domain alignment was guided by the goal of enhancing the model’s general reasoning, contextual understanding, and task adaptability, which are foundational for oncological applications. The datasets like Chain of Thought and Alpaca emphasize reasoning and logical progression, which are crucial for interpreting complex radiology impressions. These skills translate directly into the ability to parse nuanced medical terminology, infer progression or stability. OpenAssistant is the Q&A datasets that train the model to respond effectively to specific and often domain-specific queries. This capability is essential for oncological tasks where clinicians or researchers require precise answers to questions about tumor progression, diagnostic accuracy, or treatment efficacy based on imaging data.

For the medical domain, our dataset selection was as follows: (1) PubMedQA^43^ (link), which challenges models to generate answers from NIH PubMed abstracts to be compared with expert-curated responses, covering a broad spectrum of biomedical topics. (2) MedQuAD^44^ (link), drawn from 12 NIH websites, including the National Cancer Institute (NCI), provides Q&A pairs on medical subjects such as treatment, diagnosis, and side effects, along with the 2017 consumer health Q&A dataset from the National Library of Medicine (NLM). (3) MedMCQA (link), an assembly of multiple-choice questions from Indian medical school entrance exams^20^. (4) The MedQA-USMLE dataset^17^ (link), developed by Google Research to train advanced models like Med-PaLM and Med-PaLM 2.

For the oncology-specific fine-tuning process, we compiled 38,719 radiology impression notes from 3402 patients across five cancer types: lung, breast, pancreatic, prostate, and colorectal. This collection is a subset of the AACR Project GENIE Biopharma Collaborative (BPC) dataset^45,46^. These records provided detailed annotations of cancer occurrences and progression. Additionally, we gathered 600 radiology impression notes from 600 patients at UCSF, focusing on lung, breast, and prostate cancers. The UCSF radiology impression notes are distinct in format and context from those of MSK, it was only used for the cross-institutional validation to evaluate the model’s generalizability, we applied the fine-tuned oncology models trained on the MSK dataset to analyze cancer progression data from the UCSF dataset (Fig. 5a). All MSK patients included in this study were part of institutional review board (IRB)-approved research protocols (MSK IRB Protocols 12–245). Additionally, the study received independent approval from the UCSF (UCSF IRB Protocols 20–32527). Patients provided written, informed consent and were enrolled in a continuous, non-random fashion.

### Stacked alignment strategy

In this study, we trained fourteen distinct models, employing a stacked multi-turn alignment and fine-tuning process, building upon each successive model iteration. The initial phase involves aligning a foundational model, Woollie Foundation, based on the pre-trained Llama models (Fig. 1a), we employed the updated datasets Alpaca, Google’s Chain of Thought (COT) dataset, the InstructionWild dataset, and a subset of the OpenAssistant dataset (OASST1). The Woollie Foundations models were enhanced on reasoning, the chain of thoughts, and Q&A.

Subsequently, we refined our focus to the medical domain, utilizing the PubMedQA and MedQuAD datasets collected before our 2021 cut-off. This allowed us to test knowledge transfer to models predating the data used to train the baseline Llama models. We aligned our Woollie Foundation model with these datasets, resulting in the Woollie Medicine model. Further alignment was done with the recently released USMLE and MedMCQA datasets after 2021, leading to the creation of the Woollie models This stacked alignment strategy effectively addressed the challenge of “catastrophic forgetting,” which often occurs when LLMs transition between domains. We compared the Woollie models aligned using the stacked alignment strategy to those trained on a simple concatenation of all datasets (Woollia All models). The results reveal a significant performance gap, with the stacked alignment models demonstrating superior performance (Fig. 2b, Supplementary Fig. 1b). The final step involved fine-tuning Woollie with real-world oncology datasets from MSK radiology impression notes, culminating in the Woollie MSK model (Fig. 1d).

The underlying Llama models varied in size: 7B, 13B, 33B, and 65B parameters. Given the general trend that larger models perform better, we investigated this aspect by training Woollie models at 7B, 13B, 33B, and 65B. We limited the Woollie Instruction to 7B, 13B, and 33B models, while Woollie MSK was fine-tuned only at 7B and 33B sizes.

### Benchmarks

We evaluated our model using a range of benchmarks, including COQA^24^, LogiQA^22^, ARC, HellaSWAG^25^, MMLU, OpenBookQA, SciQ, PubMedQA^19^, USMLE, and MedMCQA^20^ (Supplementary Table 4). During evaluations and testing, we set the model’s generation temperature to 0.01 and top_p to 1.0 to minimize randomness and ensure deterministic behavior, as the model is being used as a classifier. Additionally, we repeated the same tasks multiple times and observed no randomness, with the performance metrics remaining constants across runs. These evaluations were conducted using a zero-shot learning approach, where the LLM is prompted with questions that generate answers without prior specific training on those questions. Typically, model performance comparisons utilize few-shot learning, where the LLM is provided with a small set of correct question-answer pairs before being asked to respond to new questions. This approach often enhances performance due to the model having relevant context. However, our model was already fine-tuned, so we aimed to ascertain its comparative improvement over the non-fine-tuned baseline Llama model using zero-shot learning. As comparisons, we incorporated 1-shot and 3-shot learning to evaluate their performance on the Woollie models (Supplementary Fig. 8). Due to the model’s context size limitation of 2048 tokens, using more than 3 shots exceeded the maximum allowable context. While we observed a performance improvement from 0-shot to 1-shot learning, further increases in the number of shots showed minimal improvement or, in some cases, a decline in performance. This decline may be attributed to the model nearing its maximum context size, causing it to lose focus on content in the middle of the sequence^47^. Further research is needed to explore the impact of larger context windows on performance.

### Privacy considerations and de-identification of clinical data for fine-tuning

The radiology impression data from the MSK patient cohort does not include protected health information (PHI). However, the impressions may refer scan events, as well as the names of referring or diagnostic physicians. To address this, we applied named entity recognition (NER) using a pre-trained BERT^48^ model with spaCy^49^. Out of 38,719 impression notes, 2467 contained physician names, which we masked as [[PERSON]]. Dates associated with scan events, considered identifiable patient data, were also replaced with [[DATE]]. Examples of de-identified radiology impressions are provided in the Supplementary Note 6.

The Woollie MSK models, trained on a non-PHI dataset, do not contain any knowledge of individual patient private information. Once downloaded to a local environment, the model can function without an external network connection, allowing it to be used on PHI data while ensuring the dataset remains secure and does not transmit outside the system. Predictions are based solely on the knowledge acquired during the model’s alignment and fine-tuning processes.

### Classification of cancer progression

A critical aspect of our testing was to assess the model’s generalizability to datasets it had not encountered during pre-training or fine-tuning. This aspect of LLM performance, especially in real-world oncology data, has not been extensively studied. The detailed breakdown of cases and patient numbers is presented (Fig. 3b, c). Through these comparative studies, we aimed to demonstrate Woollie’s proficiency in accurately interpreting and classifying oncological data from diverse institutional sources.

The radiologist has manually annotated the dataset. For the MSK dataset, we have annotated the report in two types: the detailed annotation with five labels to reflect whether the tumor has progressed or not: (a) Progressing/Worsening/Enlarging, (b) Stable/No change, (c) Improving/Responding, (d) Not stated/Indeterminate, (e) Mixed., The binary annotation combines the (a) Progressing/Worsening/Enlarging and (e) Mixed as positive case - tumor progressed (“Progressing”) and combination of other labels as tumor not progressed (“Not Progressing”). For the UCSF dataset, we only have binary annotation, with tumor progression or not.

We divided the 38,719 reports into training, validation, and testing groups for the MSK dataset, following an 80%, 10%, and 10% split, respectively. We used an optimized prompting strategy (Supplementary Note 5, Supplementary Fig. 7) with a maximum input token window of 2048 tokens, which was sufficient to accommodate the full radiology impression during training (Supplementary Fig. 9). Woollie is a generative model, and we must reformat the testing to a multiple-choice one; we prompt the radiology impression using the templates (Supplementary Note 7). The resulting fine-tuned models were Woollie MSK 7B and Woollie MSK 33B. To test their performance, we employed the Language Model Evaluation Harness framework^50^, a standard tool used across the open-source LLM community for evaluating model effectiveness. This framework was adapted to include a radiology impressions validation dataset in the evaluation criteria. It provided accuracy metrics and calculated log-likelihood, which we converted into prediction probabilities for each answer using the Softmax function. Since Woollie is a generative model, we utilized the approach described in Supplementary Note 2 to generate the AUROC, ROC curve, and the confusion matrix.

To evaluate our model’s performance across five classes, we employed the one-vs-rest strategy to calculate the AUROC. This approach involves generating an ROC curve for each class by comparing it against all other classes combined, computing the AUROC for each curve, and then averaging these values to obtain a single representative score.

In addition to AUROC, we calculated precision, recall, F1 scores, and MCC to provide a comprehensive assessment of the model’s performance (see Supplementary Table 1). AUROC was selected as a primary performance metric due to its robustness and widespread use in evaluating a model’s ability to distinguish between classes across various decision thresholds. Notably, AUROC assesses performance independently of specific classification thresholds, making it particularly useful in scenarios where these thresholds may vary. Furthermore, AUROC is effective in handling imbalanced datasets by focusing on true positive and false positive rates rather than absolute counts, providing a balanced evaluation even when class distributions are uneven.

We used the same prompt format for the UCSF dataset, consisting of 600 radiology impression notes labeled “Progression” as positive and “Not Progression” as negative. These notes underwent evaluation using the same framework with both Woollie MSK 7B and Woollie MSK 33B models. We reported on accuracy and log-likelihood measures. ROC curves were also plotted for this dataset and were directly compared with those of the MSK dataset within the exact figures (Fig. 5c).

### Cancer trajectories analysis

Instead of viewing LLM as a black box, we aim to delve into the model’s understanding of the contextual intricacies inherent in clinical narratives and oncology knowledge embedded within clinical notes. To achieve this, we thus implemented topic modeling using Woollie MSK models on the dataset of radiology impressions to garner insights into tumor progression and the pathways of cancer development, facilitating these discoveries through clustering identified topics. This approach helps us gauge the extent of cancer biology knowledge the model has assimilated during its fine-tuning phase. Employing such sophisticated topic modeling techniques is particularly beneficial in the clinical setting, where healthcare professionals are challenged to extract evolving disease patterns from a dense array of data points.

Specifically, we employed a neural topic modeling approach outlined in the study^51^. The initial step involved transforming radiology impression notes into a vectorized format using FlagEmbedding^52^, resulting in vectors of dimension 1024 for each note. To facilitate analysis, we reduced these high-dimensional vectors to 5 dimensions using Uniform Manifold Approximation and Projection (UMAP)^53^, configured with 15 neighbors for dimensionality reduction. This approach deviates from traditional k-means clustering by employing Hierarchical Density-based Spatial Clustering of Applications with Noise (HDBSCAN)^54^. HDBSCAN is adept at clustering outputs from LLMs due to its ability to manage clusters with varied densities, autonomously identify the number of clusters, and filter out noise as outlier data. This hierarchical clustering technique is precious for delving into data across different levels of detail, enabling the exploration of intricate relationships in LLM outputs. HDBSCAN is a prime tool for analyzing LLMs’ nuanced and semantically rich outputs thanks to its versatility with various distance metrics and effectiveness in handling high-dimensional data. We set the minimum cluster size to 15.

Using the Woollie MSK 33B model for summarization and topic extraction, we focused on identifying the top 10 most relevant keywords for each note, restricting the analysis to 20 topics with the highest probabilities. The topic extraction was first applied to individual cancer types (Fig. 6a–e), and then extended to encompass all notes, identifying all pertinent topics (Fig. 6f). We visualized the topics using UMAP to reduce dimensions to two, with each color representing the same topics from the Woollie model. The size of the dots indicates the number of radiology impression notes within that reduced dimensional representation.

Considering the complexity of the large language model and its potential clinical applications, we conducted explainability studies using LIME (Supplementary Note 3). Combining insights from LLM-based topic modeling and LIME explanations highlights the knowledge acquired by the Woollie models and bolsters confidence in their explainability for clinical use. While LLMs excel in extracting high-level concepts through topic modeling, LIME effectively emphasizes the importance of local features, providing a complementary perspective on the model’s interpretability.

The Sankey plot (Fig. 6g) visually represents the disease trajectory across different cancer sites within patients from diagnosis onward. This plot offers insightful visual analytics on how patients’ cancer types may evolve or spread to other sites over time, highlighting potential metastasis pathways or indicating typical trajectories in disease progression.

## Supplementary information

Supplementary information

## Data Availability

The radiology impression data used in this study is part of our MSK-CHORD project (“Jee, J., Fong, C., Pichotta, K. et al. Automated real-world data integration improves cancer outcome prediction. *Nature* 636, 728–736 (2024). 10.1038/s41586-024-08167-5”). The labeled data is publicly available via cBioPortal at MSK-CHORD 2024 and GENIE cBioPortal. Additionally, it is included in the GENIE BPC Cohort, with annotated labels accessible at AACR Project GENIE Data, which provides instructions for downloading the data. The Woollie Foundation, Woollie Medicine, and Woollie Models are available on Hugging Face at Hugging Face - Woollie Collection. The collection includes models with parameters ranging from 7B to 65B. The original radiology impression notes cannot be shared with researchers outside of the study team approved by the IRB of the participating institution due to MSK legal policies. Access to anonymized data can be shared through a data transfer agreement (DTA) managed by the principal institution. Woollie MSK 7B and 33B models trained on MSK clinical datasets can be shared upon legal agreement, subject to appropriate review and approval.

## Code availability

Alignment, finetuning and measurement codes for this study are available at https://github.com/msk-mph/woollie.
