# Supplementary material for: Large language model trained on clinical oncology data predicts cancer progression
Source: NPJ Digit Med. 2025 Jul 2;8:397. doi: 10.1038/s41746-025-01780-2 (PMC12223279; doi:10.1038/s41746-025-01780-2)
Supplement: Supplementary file 1 — Supplementary information [file 41746_2025_1780_MOESM1_ESM.pdf]

Supplementary Information

Supplementary Notes 3

Supplementary Note 1: Woollie models architectures and loss function for fine-tuning 3

Supplementary Note 2: Computation of AUROC for the generative model 3

Supplementary Note 3: Explosibility of the Woollie models for cancer progressing 5

Supplementary Note 4: Alignment and fine-tuning parameters 5

Supplementary Note 5: Prompts design and its dependence on model performance. 6

Supplementary Note 6: MSK radiology impression samples with masked identified information and UCSF radiology impression sample 9

Supplementary Note 7: Classification of cancer progression 10

Supplementary References 10

Supplementary Figures 12

Supplementary Figure 1: Evaluation of Woollie models on non-medical and medical domain tests 12

Supplementary Figure 2: ROC curves of LLM models on the MSK data performance on each cancer type 14

Supplementary Figure 3: ROC curves of LLM models on the UCSF data performance on each cancer type 16

Supplementary Figure 4: LIME analysis of the explainability of Woollie MSK 7B models for cancer progression classification. 18

Supplementary Figure 5: LIME analysis of the explainability of Woollie MSK 33B models for cancer progression classification. 20

Supplementary Figure 6: Comparative performance of leading open-source LLM model on cancer progression dataset against Woollie models 22

Supplementary Figure 7: Influence of prompt type on model performance. 24

Supplementary Figure 8: The impact of few-shot learning on Woollie's performance 26

Supplementary Figure 9: Token distribution of the MSK radiology impression dataset 28

Supplementary Figure 10: Woollie architecture diagram 30

Supplementary Tables 32

Supplementary Table 1: List of metrics for models in this study 32

Supplementary Table 2: Model resource consumption and inference time 33

Supplementary Table 3: Alignment and fine-tune hyper-parameters for all Woollie models 34

Supplementary Table 4: Selection of benchmarks utilized for evaluating our alignment and fine-tuning approaches 35

Supplementary Table 5: Comparison of model performance across sociodemographic groups and cancer types. 36

Supplementary Notes

Supplementary Note 1: Woollie models architectures and loss function for fine-tuning

Woollie models are built on the META Llama 1 architecture, a variation of the transformer model. These generative models support a maximum input context of 2048 tokens and are available in versions with 7B, 13B, 33B, and 65B parameters. While we provide a brief overview here, detailed specifications can be found in META's original publication[1]. A simplified architecture diagram is presented in Supplementary Figure 10.

Compared to the original transformer architecture, Llama introduces several key differences: (a) RMS (Root Mean Square) Normalization**:** Instead of layer normalization, Llama employs RMS normalization after each multi-head attention block. This approach involves centering values around zero and scaling them to a standard deviation of one, ensuring a normalized distribution of values for improved model stability and performance. (b) Rotary Positional Embedding**:** Llama uses rotary positional embeddings, which apply rotation operations to positional encodings. This dynamic approach allows the model to learn positional representations during training instead of relying on static, pre-calculated positional vectors. (c) SwiGLU Activation Function**:** In the feed-forward layers, Llama uses the SwiGLU activation function instead of the traditional ReLU, enhancing its ability to model complex relationships in the data.

During alignment and fine-tuning of Woollie models, the training dataset includes inputs and their corresponding expected outputs. Inputs are fed into the model, which generates logits for the next tokens. The loss function is then computed as the summation of the cross-entropy loss for all tokens in each sequence. This approach enables the model to learn the desired behavior effectively.

$$F[\emptyset] = -\sum_{i=1}^{I}\sum_{t=1}^{T_i}\log\left[\Pr\left(y_{i,t+1}\middle|\boldsymbol{x}_i, y_{i,1\cdots t,}\emptyset\right)\right]$$

The target tokens for the ith prompt by $\boldsymbol{x}_i = \left[x_{i,1}, x_{i,2}, \cdots\right]$ and the tokens in the corresponding response $\boldsymbol{y}_i = \left[y_{i,1}, y_{i,2}, \cdots y_{i,T_i}\right]\emptyset$ represent the model parameters, which will be updated during backpropagation. In the fine-tuning, we don't update all parameters; we use Low-Rank Adaptation[2], and only a fraction of the parameters will be updated during the backpropagation.

Supplementary Note 2: Computation of AUROC for the generative model

The performance of the fine-tuned model is evaluated by generating receiver operating characteristic (ROC) curves. To compute the true positive rates (TPRs) and false positive rates (FPRs), the model's predictions are scored by calculating the log-likelihoods of label sequences given their respective input prompts.

For each label, a unique input prompt is generated by appending the label to a shared base prompt. For example, if there are five possible labels, five separate prompts are created, each ending with

one of the five labels. The model processes each prompt to compute the likelihood of the appended label, as follows:

1. The input text is tokenized, and padding is applied to prepare it for the model.
2. The tokenized input is passed through the model, and logits corresponding to the label tokens are extracted.
3. The log-likelihood of the label sequence is computed from the logits using the softmax function, which converts the logits into probabilities.

The log-likelihoods are then used to score the labels, facilitating the computation of TPRs and FPRs for various decision thresholds. These metrics are ultimately used to generate the ROC curves, which provide a comprehensive evaluation of the model's classification performance.

The mathematical representation of step 3 is given as follows. Let $L_{\text{logits}} = [\text{logit}_1, \text{logit}_2, \ldots, \text{logit}_n]$ represent the output from the model, where each logit_i corresponds to the raw score, i.e., logits for a token in the sequence and $L_{\text{labels}} = [\text{label}_1, \text{label}_2, \ldots, \text{label}_n]$ represent the token ID of the label sequence that is being evaluated. The raw unnormalized scores are converted to probabilities using,

$$P(\text{token}_i) = \frac{e^{\text{logit}_i}}{\sum_j e^{\text{logit}_j}}, (1)$$

where logit_i is the logit for token_i, and the denominator is the sum of exponentiated logits over all possible tokens. The next step is computing the log-probabilities by applying the log function to the softmax values, which is described in eq. (2).

$$\log\big(P(\text{token}_i)\big) = \log\left(\frac{e^{\text{logit}_i}}{\sum_j e^{\text{logit}_j}}\right) = \text{logit}_i - \log\left(\sum\nolimits_j e^{\text{logit}_j}\right), (2)$$

For a sequence of label tokens, the log-likelihood is the sum of the log-probabilities for each label token ID, i.e., label_k.

$$\text{log-likelihood} = \sum_{k=1}^{m} \log\big(P(\text{label}_k)\big) \ldots (3)$$

where $P(\text{label}_k)$ is the probability of the kth label token and is computed using the log-softmax function. Combining equations (2) and (3), we get,

$$\text{log-likelihood} = \sum_{k=1}^{m} \left[logit_{label_k} - log\left(\sum\nolimits_j exp\big(logit_j\big)\right)\right], (4)$$

This equation sums the log-probabilities for each label token, producing the total log-likelihood for the sequence of labeled tokens.

Supplementary Note 3: Explosibility of the Woollie models for cancer progressing

To compare the topic modeling approach using Woollie for extracting global features, we conducted an additional study with local interpretable model-agnostic explanations (LIME) to analyze how the model interprets topics and how local features contribute to the final classification.

We used the LIME Python package (link) and configured it with a feature size of 10 and a sample size of 100. However, due to the package's limitations, we were unable to directly test LLMs using GPU clusters with job queuing and management. Instead, we generated 100 perturbed samples of input data using LIME and submitted them as batch jobs to the Woollie model. The model returned probability predictions for each labeled impression note, which were then passed back into LIME for final analysis of the importance of local features.

The results for Woollie MSK 7B and MSK 33B models are presented in Supplementary Figure 4 and Supplementary Figure 5, respectively. We observed some interesting differences by comparing the LIME-generated outputs with the LLM-based topic modeling in our study. LIME perturbs the input by randomly removing or replacing words to create samples. These perturbed samples are processed by Woollie, which assigns probabilities to each class, "Not Progressing" or "Progressing." In contrast, the topic modeling extracts global features rather than focusing on word-level perturbations.

LLM-based topic modeling identified broader knowledge-level concepts, such as "liver metastasis" or "right frontal lobe lesion." On the other hand, LIME highlighted the importance of local features, such as individual words like "increasing," "small," "metastasis," or "FDG." Notably, in the Woollie MSK 7B model, the word "FDG" played a significant role in classifying cases as "Progressing." FDG (fluorodeoxyglucose) is a radiotracer commonly used in oncology imaging, particularly PET scans, to detect tumors. Since tumors exhibit increased glucose metabolism, FDG accumulates in tumor cells, signaling active tumor progression. The model's understanding of FDG as a progression marker demonstrates its ability to effectively learn clinical concepts.

Similarly, in the Woollie MSK 33B model, the classification of "Progressing" cases was strongly influenced by the "increase" in tumor size, aligning with radiologists' assessments. Conversely, in "Not Progressing" cases, the word "small" was a significant negative indicator, again consistent with human interpretation.

Combining the insights from LLM-based topic modeling and LIME explanations highlights the knowledge learned by the Woollie models and strengthens confidence in their explainability for clinical use. While LLMs excel at extracting high-level concepts, LIME effectively demonstrates the importance of local features, offering a complementary perspective on the model's interpretability.

Supplementary Note 4: Alignment and fine-tuning parameters

There are three prominent fine-tuning methods: (i) full parameter fine-tuning, (ii) partial layer fine-tuning, and (iii) Parameter-Efficient Fine-Tuning (PEFT). While methods (i) and (ii) require substantial GPU memory, PEFT offers an efficient alternative, achieving comparable performance to full parameter fine-tuning but dramatically reducing memory usage. This efficiency allows application across all four base models: 7B, 13B, 33B, and 65B.

For the Woollie models (7B, 13B, 33B), we employed a PEFT with Low Rank[2] adaptation for fine-tuning, using rank $r = 8$, $\alpha = 16$, and 0.05 dropout settings. The fine-tuning targeted three weights in self-attention layers (W_k, W_q, W_v) and three linear MLP layers ($W_{up}, W_{down}, W_{gate}$), accounting for 0.26%, 0.21% and 0.18% of the total fine-tuning parameters. We maintained the input context size at a maximum of 2048 tokens, the same as the pre-trained Llama models. It is the maximum context window of the underlying models that have been trained. Despite the possibility of using DeepSpeed ZeRO[3] to federate the model, we opted for a single GPU approach (A100 80GB) due to its sufficiency for our chosen model sizes, intercommunication in the cluster, and our strategy of data parallelization (we used DDP from PyTorch) and gradient accumulation to minimize memory consumption while maintaining a global batch size of 128. Techniques like gradient accumulation, bfloat16, and gradient checkpointing were utilized to further reduce per-GPU memory usage. The maximum number of training epochs was set to 3. We also configured the system to save model weights every 200 steps and record logs at 10-step intervals for detailed tracking and analysis.

For the largest Woollie models with 65 billion parameters, we switched to QLoRA (Quantized Low-Rank Adaptation) with 4-bit NormalFloat and double quantization, supplemented by paged optimizers[4]. The settings used $r = 64, \alpha = 16$ and 0.05 dropout, with adaptation to four weights in self-attention (W_k, W_q, W_v, W_o), and three linear MLP layers ($W_{up}, W_{down}, W_{gate}$). The total trainable parameters comprised 3.92% of the entire parameter model. Unlike the original QLoRA approach of using a single GPU, we implemented multiple data parallelization across several GPUs. This parallelization significantly accelerated the training process for larger datasets, with a global batch size of 64 for the 65B models (See Supplementary Table 3 for the full parameters).

Supplementary Note 5: Prompts design and its dependence on model performance.

The prompt dependency of LLMs during inference is well-documented, yet the extent of this influence across various prompt types during alignment or fine-tuning remains less well-explored. We conducted a comparative analysis utilizing two distinct prompt types during the alignment and fine-tuning stages: Instruction prompts and Chat prompts.

Instruction prompts provide context and a question, enabling the model to generate a single, focused response against a known standard. They are beneficial for teaching the model to respond with specific information. Chat prompts, conversely, are designed as multi-turn dialogues, prompting the model to predict subsequent lines based on the preceding text. This iterative process allows the LLM to learn and refine its ability to maintain conversations across various topics.

To facilitate comparative analysis, we aligned three models—Woollie Instruction 7B, 13B, and 33B—using an Instruction prompt template akin to Alpaca's format. The template, highlighted

below, employs placeholders enclosed in curly brackets for dynamic content substitution during training. Subsequently, the complete prompt undergoes tokenization via a tokenizer.

```
Below is an instruction that describes a task. Write a response that appropriately completes the request.
### Instruction:
{instruction}
### Response:
{response}
```

For a sample from the PubMed dataset:

```
Below is an instruction that describes a task. Write a response that appropriately completes the request.

### Instruction:
Describe how the genetic code is read and translated in the synthesis of a protein?

### Response:
The genetic code is the set of rules that defines how the information encoded within DNA and RNA is translated into proteins by living cells. In the synthesis of a protein, the genetic code is read and translated in several steps.

First, the DNA molecule, which contains the genetic information, is transcribed into a molecule of messenger RNA (mRNA). This process takes place in the nucleus of the cell and is carried out by an enzyme called RNA polymerase. During transcription, the RNA polymerase reads the DNA code and synthesizes a complementary mRNA molecule based on the sequence of nucleotides in the DNA.

Next, the mRNA molecule is transported out of the nucleus and into the cytoplasm, where it is translated into a protein by ribosomes. The ribosomes read the mRNA sequence in groups of three nucleotides, called codons. Each codon codes for a specific amino acid, which is the building block of proteins.

The translation process begins when the ribosome binds to the start codon on the mRNA molecule. The ribosome then moves along the mRNA, reading each codon and adding the corresponding amino acid to the growing protein chain. This process continues until the ribosome reaches a stop codon, which signals the end of translation.

The newly synthesized protein then undergoes further processing and folding before it becomes functional. Overall, the process of reading and translating the genetic code is a complex and highly regulated process that is essential for the proper functioning of the cell and the organism as a whole.
```

We noted that specific datasets comprised multi-turn conversations that were unsuitable for the Instruction prompt format. We employed a Chat prompt format to accommodate these, akin to the one referenced in [5]. As with the Instruction prompt, the template portions are highlighted in this format. These prompts consistently conclude with \n[|Human|], signaling the model to respond conversationally and prompting human input.

> The conversation between human and AI assistant.\n[|Human|] {input[0]}\n[|AI|] {response[0]}\n[|Human|] {input[1]}\n[|AI|] {response[1]}\n[|Human|]

For Chat prompt, one example from MedQuAD:

> The conversation between human and AI assistant.\n[|Human|] I'd like to know if there are any safety concerns or special precautions about using Palm Oil.\n[|AI|] Yes, there are some concerns about the environmental impact and potential health risks associated with Palm Oil. Would you like more information on this topic?\n[|Human|] Yes, please tell me more about the concerns surrounding Palm Oil.\n[|AI|] One concern is deforestation and the impact it has on the habitats of endangered species, such as orangutans. Additionally, the use of heavy pesticides and fertilizers in Palm Oil production can be harmful to the environment and local communities. Finally, the high levels of saturated fats present in Palm Oil can contribute to health issues if consumed in excess. These are just a few concerns surrounding the use of Palm Oil.\n[|Human|] Are there any alternative oils that can be used instead of Palm Oil?\n[|AI|] Yes, there are several alternative oils, including canola oil, sunflower oil, and soybean oil. These alternatives have their own sets of benefits and drawbacks, so it's important to choose the best option based on the specific needs of the product or recipe.\n[|Human|]

Across non-medical benchmarks, both Instruction and Chat-prompted models delivered performances aligned with the baseline Llama models(Supplementary Figure 7). However, the Chat-prompted versions exhibited a marked improvement over the Instruction-prompted Woollie models. For example, in the MMLU benchmark, the Woollie 13B Chat model achieved a score of 0.35, surpassing both the Woollie Instruction 13B at 0.32 and the Llama 13B at 0.33, with a statistically significant difference ($p < 0.001$).

In the realm of medical benchmarks, Chat-prompt-aligned Woollie models clearly outperformed the Instruction-prompt-aligned versions. Averaging accuracy scores across PubMedQA, MedMCQA, and USMLE, the Woollie models at 7B, 13B, and 33B scored 0.46 versus 0.44, 0.52 versus 0.49, and 0.58 versus 0.53 respectively when compared to Woollie Instruction models, each with significant differences ($p < 0.001$). Additionally, when aggregating results from all evaluated benchmarks, comparisons between Woollie and Woollie Instruction models at the 7B, 13B, and 33B levels showed scores of 0.48 versus 0.46, 0.53 versus 0.50, and 0.57 versus 0.54 respectively, again highlighting significant improvements ($p < 0.001$).

The Chat-prompt-aligned Woollie variants matched and surpassed the baseline Llama models in general and medical-specific benchmarks. In contrast, the Woollie Instruction models displayed slightly lower performance than the Llama baseline in non-medical tests, underscoring the significant impact of prompt type on model alignment and the effectiveness of fine-tuning. These insights have led us to prefer Chat prompts in our model alignment processes.

Supplementary Note 6: MSK radiology impression samples with masked identified information and UCSF radiology impression sample

The radiology impression part of the radiology reports does not include the patient protected health information (PHI), and sometimes it includes referral physician's name. We used BERT for NER and scanned all 3871 impression notes, we found 2467 notes include the physician's names, we masked that as [[PERSON]]. Another patient-related identifier is event date; we masked it as [[DATE]]. See below the examples from MSK radiology impression data for training with masked physician names and event data.

MSK radiology impression sample

> 1. Since [[DATE]], no substantial change in two right lower lobe \nnodules. Stable left basilar punctate nodular opacity. No new pulmonary \nnodules. \n2. New hypoattenuation of the right hepatic lobe, probably reflecting \npostradiation changes.\nPreliminary findings were discussed with Dr. [[PERSON]] on Thoracic Service on \n[[DATE]]

> 1. Since [[DATE]], decreased size of right perihilar, and right \nlower lobe masses consistent with tumor.\n2. Decreased thoracic nodal metastases.\n3. Resolution of hepatic metastases. Stable lesion in hepatic segment \n2/3, probably hemangioma.\n4. Increased size of splenic mass with decreased peripheral soft tissue \nrim, suspicious for necrotic metastasis.\n5. Decreased size of some of bilateral renal masses suspicious for \nmetastases.\n6. Increased size and sclerosis of osseous metastases, probably due to \nhealing in light of decreased metastases at other sites.\n7. Increased groundglass opacities in the right lung, possibly \ninflammatory, infectious.\n \nDictatedBy: [[PERSON]], MD\nStaff Radiologist:[[PERSON]], MD\n \nI attest that the above IMPRESSION is based upon my personal examination of\nthe entire imaging study and that I have reviewed and approved this report.

The UCSF dataset was used exclusively for validation purposes, and its impression notes include PHI. However, there were no PHI-related concerns since the testing was conducted on locally deployed Woollie models within the UCSF firewall. It is worth noting that the UCSF radiology impression format was not encountered during Woollie's training. For reference, we provide a sample impression with PHI removed.

1. Low-level diffuse FDG avidity and contrast enhancement associated with the lateral left breast, this corresponds to the findings of *********** enhancement on the breast MRI and is suspicious for residual metabolically active malignancy. 2. Small left axillary nodes have decreased in size from */**/**** and are no longer pathologically enlarged and demonstrate no FDG avidity. No FDG avid metastatic disease. 3. A few stable lung nodules measuring up to 5 mm. Follow-up per oncologic protocol. This study was reviewed by Dr. ***** ******* of Nuclear Medicine. . END OF IMPRESSION

Supplementary Note 7: Classification of cancer progression

Since Woollie is a generative model, we must reformat the testing as multiple choice; we prompt the radiology impression with the following template and ask the LLM to select the following options from the five labels.

Radiology Impression: {impression} \nPlease provide a most likely assessment of the tumor's progression in the following choices?\nChoices:\n (A) Progressing/Worsening/Enlarging\n(B) Stable/No change\n(C) Improving/Responding\n(D) Not stated/Indeterminate\n(E) Mixed\n Answer:

For example

Radiology Impression: 1. Since July 31, 2012 hepatic metastasis has decreased and is barely \nperceptible.\n2. Stable necrotic pancreatic body mass.\n3. Decrease in retrocrural, upper abdominal and retroperitoneal \nlymphadenopathy \nPlease provide a most likely assessment of the tumor's progression in the following choices?\nChoices:\n (A) Progressing/Worsening/Enlarging\n(B) Stable/No change\n(C) Improving/Responding\n(D) Not stated/Indeterminate\n(E) Mixed\n Answer:

For the UCSF dataset, we adapted the same conversational prompt format. We reported on accuracy and log-likelihood measures. ROC curves were also plotted for this dataset and directly compared with the MSK datasets.

Supplementary References

1 Touvron, H. *et al.* Llama: Open and efficient foundation language models. *arXiv preprint arXiv:2302.13971* (2023).

2 Hu, E. J. *et al.* Lora: Low-rank adaptation of large language models. *arXiv preprint arXiv:2106.09685* (2021).
3 Rajbhandari, S., Rasley, J., Ruwase, O. & He, Y. in *SC20: International Conference for High Performance Computing, Networking, Storage and Analysis.* 1-16 (IEEE).
4 Dettmers, T., Pagnoni, A., Holtzman, A. & Zettlemoyer, L. Qlora: Efficient finetuning of quantized llms. *arXiv preprint arXiv:2305.14314* (2023).
5 Xu, C., Guo, D., Duan, N. & McAuley, J. Baize: An Open-Source Chat Model with Parameter-Efficient Tuning on Se lf-Chat Data. *arXiv preprint arXiv:2304.01196* (2023).

Supplementary Figures

Supplementary Figure 1: Evaluation of Woollie models on non-medical and medical domain tests

a), Polar plot comparing accuracy on standard non-medical and medical benchmarks between Llama and Woollie models. The shaded areas highlight significant performance improvements in the medical domain achieved by the Woollie models. **b),** The stacked alignment strategy effectively mitigates catastrophic forgetting in LLMs. Comparisons across models of varying sizes (7B, 13B, 33B, and 65B) were conducted among Llama, Woollie Foundation, Woollie Medicine, Woollie, and Woollie All models. While modest improvements were observed in standard non-medical benchmarks for reasoning and logic, significant performance gains were achieved in the medical domain. In contrast, the non-stacked aligned Woollie All models demonstrated a performance gap across all tests when compared to the stacked Woollie models. **c),** A scaling study mapped the performance of Llama, Woollie Foundation, and Woollie models across model sizes from 7B to 65B parameters over 11 tests. The findings indicate that larger models typically deliver better performance. Unlike the baseline Llama model, which did not exhibit this trend in the PubMedQA test, all aligned models demonstrated improved scaling behaviors post-alignment.

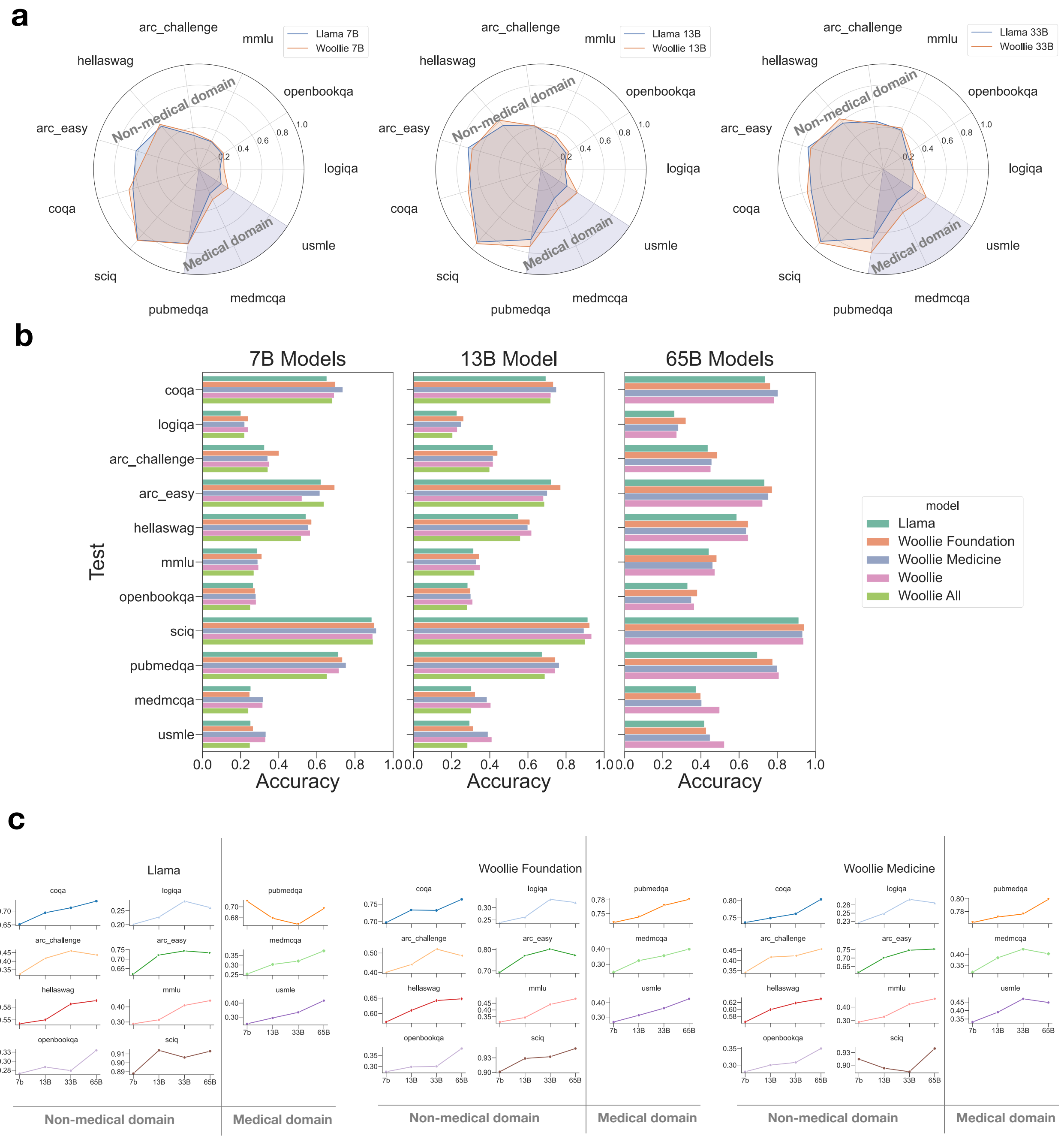
a
arc_challenge
mmlu
hellaswag
openbookqa
arc_easy
logiqa
coqa
usmle
sciq
medmcqa
pubmedqa
Non-medical domain
Medical domain
Llama 7B
Woollie 7B
Llama 13B
Woollie 13B
Llama 33B
Woollie 33B
b
7B Models
13B Model
65B Models
Test
Accuracy
model
Llama
Woollie Foundation
Woollie Medicine
Woollie
Woollie All
c
Llama
Woollie Foundation
Woollie Medicine
Non-medical domain
Medical domain

Supplementary Figure 2: ROC curves of LLM models on the MSK data performance on each cancer type

a), On the left, the Llama 7B model's ROC curves and confusion matrices for breast, colorectal, lung, prostate, and pancreatic cancers, alongside its aggregated ROC curves, are displayed. On the right, the Woollie MSK 7B model, fine-tuned using the MSK dataset, showcases its ROC curves and comprehensive performance metrics. This illustrates how alignment with general medical and oncology domains can substantially enhance LLM efficacy. The Woollie MSK 7B models excel across all cancer types, with standout performances in colorectal and pancreatic cancers. **b)**, Employing the same test dataset, we evaluated the 33B models' performance among Llama, Woollie, and Woollie MSK versions. The Woollie MSK 33B model demonstrates an outstanding 0.97 AUROC overall, with an exceptionally remarkable 0.98 AUROC in pancreatic cancer progression. Left: Llama, Right: Woollie MSK

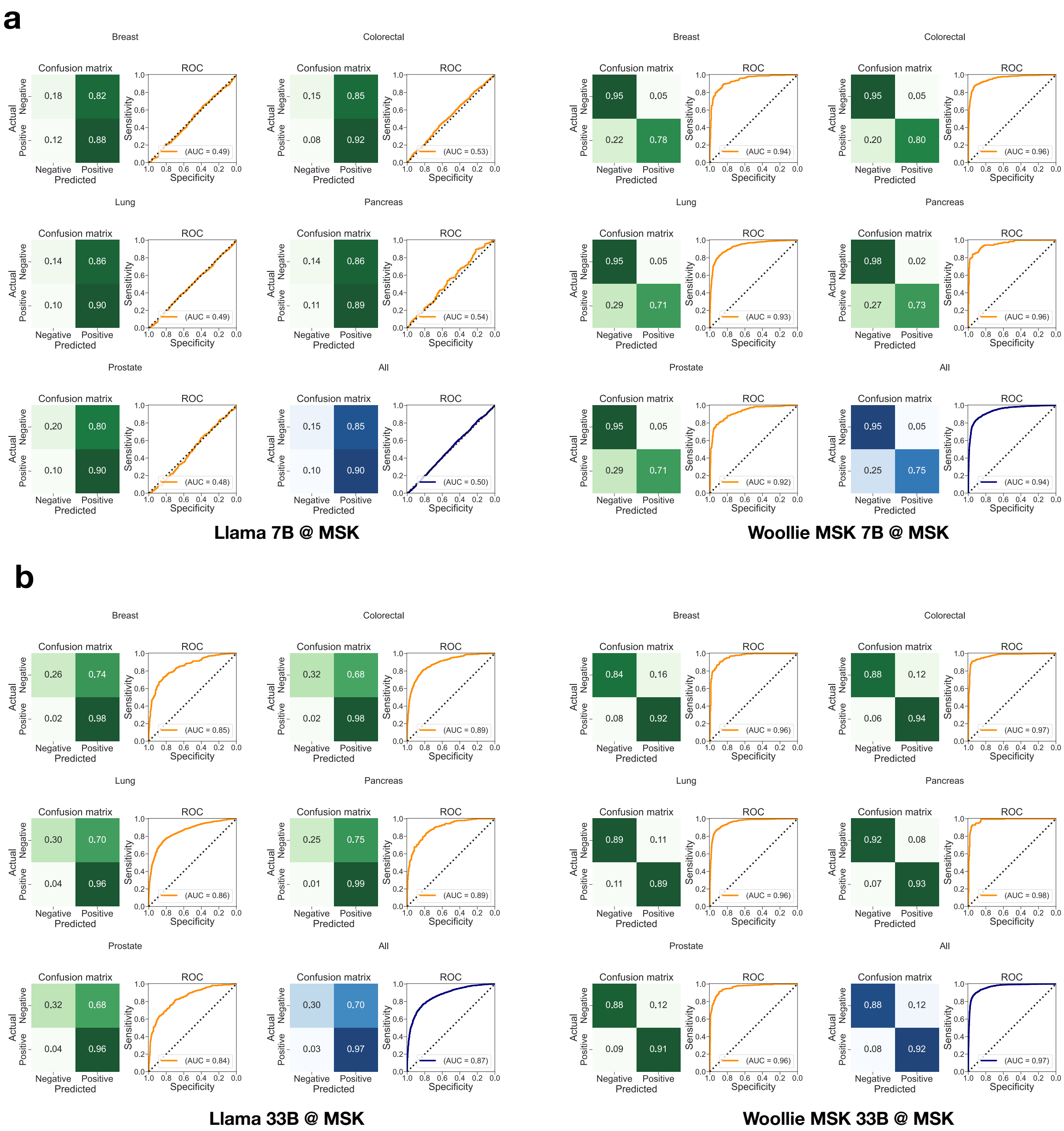
a
Breast
Colorectal
Lung
Pancreas
Prostate
All
Confusion matrix
ROC
Actual
Predicted
Sensitivity
Specificity
Llama 7B @ MSK
Woollie MSK 7B @ MSK
b
Llama 33B @ MSK
Woollie MSK 33B @ MSK

Supplementary Figure 3: ROC curves of LLM models on the UCSF data performance on each cancer type

a), The Woollie MSK 7B model, optimized with the MSK dataset and validated against the UCSF dataset, is featured with its ROC curves and a complete performance overview. This configuration demonstrates the successful transfer of oncology knowledge, even with previously unseen datasets, from one institution to another. The Woollie MSK 7B models stand out in their performance across all examined cancer types, particularly in lung cancer. **b)**, The performance of Woollie MSK 33B model was assessed using the identical test dataset. The model achieved an impressive overall AUROC of 0.88, with an exceptional 0.95 AUROC in lung cancer.

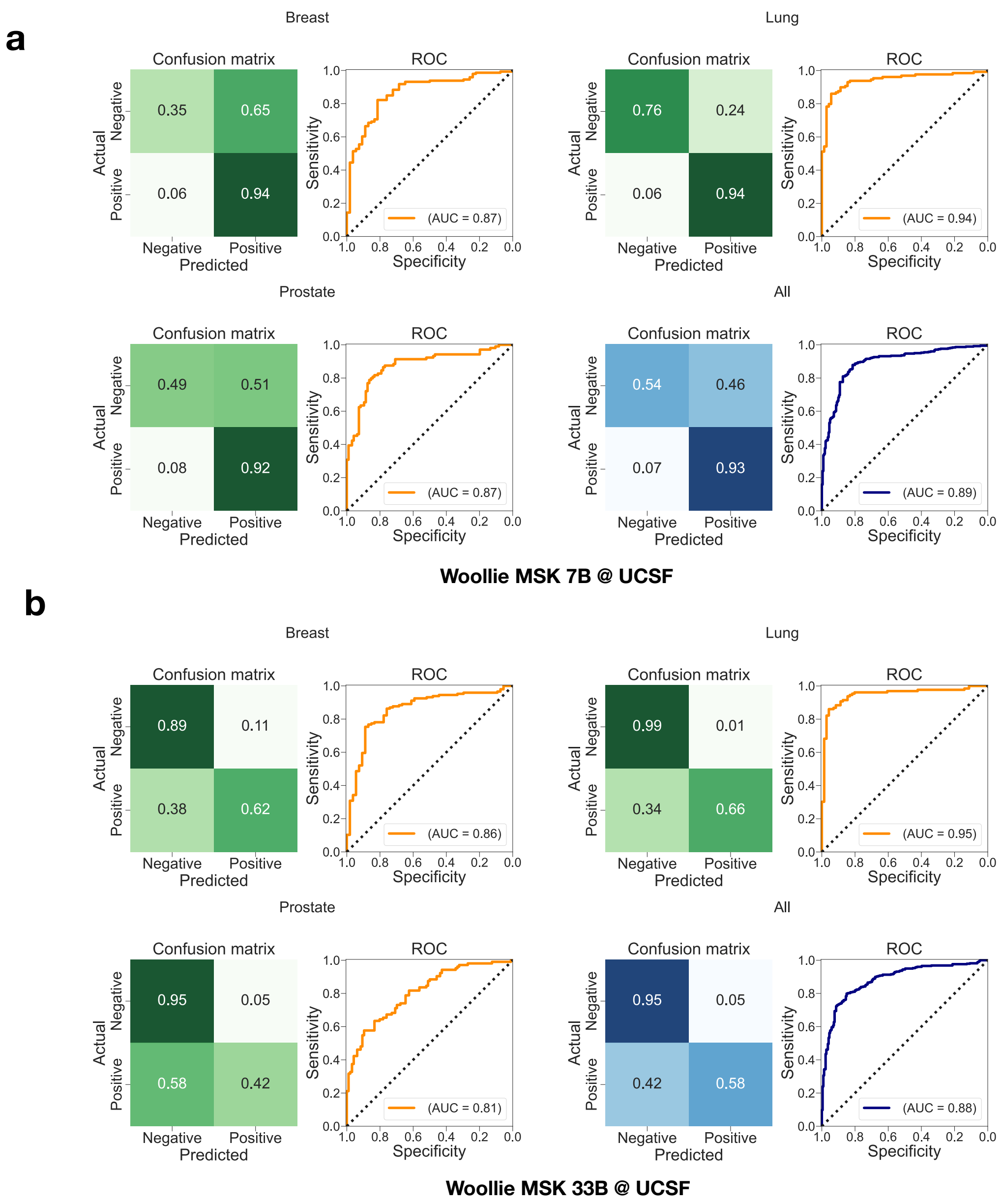
a
Breast
Lung
Prostate
All
Confusion matrix
ROC
Actual
Negative
Positive
Predicted
Sensitivity
Specificity
0.35
0.65
0.06
0.94
(AUC = 0.87)
0.76
0.24
0.06
0.94
(AUC = 0.94)
0.49
0.51
0.08
0.92
(AUC = 0.87)
0.54
0.46
0.07
0.93
(AUC = 0.89)
Woollie MSK 7B @ UCSF
b
Breast
Lung
Prostate
All
0.89
0.11
0.38
0.62
(AUC = 0.86)
0.99
0.01
0.34
0.66
(AUC = 0.95)
0.95
0.05
0.58
0.42
(AUC = 0.81)
0.95
0.05
0.42
0.58
(AUC = 0.88)
Woollie MSK 33B @ UCSF

Supplementary Figure 4: LIME analysis of the explainability of Woollie MSK 7B models for cancer progression classification.

Local interpretable model-agnostic explanations (LIME) perturbs input data by randomly removing or replacing words to create samples. These perturbed samples are processed by Woollie, which assigns probabilities to each class, “Not Progressing” or “Progressing.” For the “Not Progressing” case, the word “mild” significantly contributed to the prediction. Conversely, in the “Progressing” case, the word “FDG” played a pivotal role in the classification. FDG (fluorodeoxyglucose) is a radiotracer commonly used in oncology imaging, particularly PET scans, to detect tumors. As tumors exhibit increased glucose metabolism, FDG accumulates in tumor cells, signaling active tumor progression. The model's recognition of FDG as a marker of progression demonstrates its ability to effectively learn and apply clinical concepts.

Woollie MSK 7B

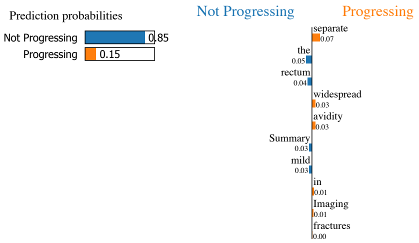

Text with highlighted words

Since [[DATE]],

1. Stable mild FDG-avidity in the axial and appendicular skeleton.
2. New mild FDG-avidity in the sigmoid colon and rectum.
3. CT findings are described in a separate report from the same date.

[[DATE]] Integrated Imaging Summary for recent CT and PET-CT
scans
RECENT STUDY: CT performed [[DATE]]
SUMMARY:
1. Stable mild FDG-avidity associated with widespread sclerotic osseous
lesions compared with [[DATE]]. The intensity of FDG-avidity
appears visually decreased compared with outside hospital PET/CT dated
[[DATE]].
2. New mild FDG-avidity in the sigmoid colon and rectum, consistent with
reported history of Crohn's disease.
3. Posttraumatic fractures in the left transverse processes of the lumbar
spine.

True : Not Progressing

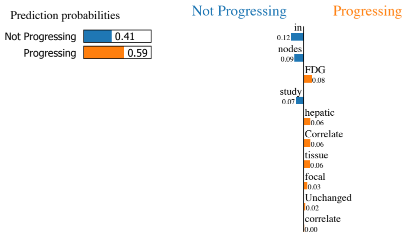

Text with highlighted words

Since [[DATE]],
1. New and increased FDG avid bilobar hepatic metastases.
2. Unchanged FDG avid scattered pelvic lymph nodes, suspicious for
metastases.
3. Unchanged non-FDG avid subcentimeter left pulmonary nodules,
suspicious for metastases.
4. Persistent FDG focal activity in the prostate, possibly represent
disease. Correlate clinically and consider tissue correlate.

Integrated Imaging Summary for above study and CT chest, abdomen and
pelvis performed [[DATE]]

SUMMARY: Concordant findings.
1. New and increased FDG avid bilobar hepatic metastases.
2. Unchanged FDG avid scattered pelvic lymph nodes, suspicious for
metastases.
3. Unchanged non-FDG avid subcentimeter left pulmonary nodules,
suspicious for metastases.
4. Persistent FDG focal activity in the prostate, possibly represent
disease. Correlate clinically and consider tissue correlate.

True : Progressing

Supplementary Figure 5: LIME analysis of the explainability of Woollie MSK 33B models for cancer progression classification.

Similar to the Woollie MSK 7B model, we analyzed the local features of the Woollie MSK 33B model. For a “Not Progressing” case, the word “small” significantly contributed to the prediction. In contrast, for a “Progressing” case, the word “increased” played a pivotal role in the classification. This demonstrates the model's ability to understand and leverage word-level features to make accurate predictions.

Woollie MSK 33B

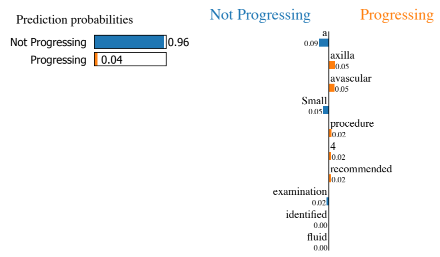

Text with highlighted words

1. Since [[DATE]], previous right upper lobe pulmonary FDG avid
nodule is unchanged and suspicious for primary lung neoplasm.

2. Previous FDG avid adenopathy is again demonstrated at the right
supraclavicular, mediastinal, right hilar, and left axillary areas.

3. Small focal fluid collection at the left axilla consistent with recent
procedure. Skin thickening of the left breast is identified, and although
possibly related to procedure, the possibility of an inflammatory
carcinoma the left breast has a possible consideration. Correlation with
clinical examination is recommended.

4. Small focal radiolucency with sclerotic margin without contour
deformity at the left femoral head is consistent with mild avascular
necrosis.

5. Moderate emphysema

True : Not Progressing

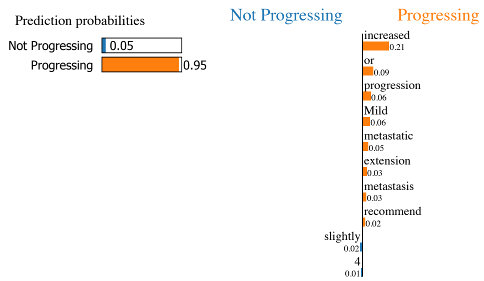

Text with highlighted words

1. Since [[DATE]], mixed interval changes in widespread mixed,
predominantly sclerotic, osseous metastases with some lesions unchanged
and some with increased sclerotic or increased lytic components. Findings
suspicious for progression of disease but recommend correlation with
same-day bone scan. Mild spinal canal extension at several upper to mid
lumbar levels unchanged to slightly increased in size; MRI can be
considered for further evaluation, as clinically indicated.
2. Scattered bilateral subcentimeter pulmonary nodules, some unchanged
and some increased in size or conspicuity, possibly metastatic.
3. Slightly increased size of hyperdense aortocaval lymph node suspicious
for metastasis.
4. Increased conspicuity of nonspecific too small to characterize
hypodense lesion in hepatic segment 6; recommend follow-up.

True : Progressing

Supplementary Figure 6: Comparative performance of leading open-source LLM model on cancer progression dataset against Woollie models

a), The Guanaco 65B model, developed by refining the Llama 65B model, achieved a commendable 99% of ChatGPT's benchmark performance, leading the pack in the open-source LLM space. Its performance was assessed using the MSK oncology dataset on each cancer type, where it recorded an overall AUROC of 0.83. **b)**, When evaluated on a dataset categorized into five labels, the Guanaco 65B model's micro-average AUROC stood at 0.75. **c)**, Despite the Guanaco 65B model's impressive 65 billion parameters—nine times that of a 7 billion parameter model—its effectiveness of AUROC lagged behind our Woollie MSK 7B model. Moreover, it was outperformed by the Woollie 33B model, which was only fine-tuned on general medical domain data. A darker background color has better performance.

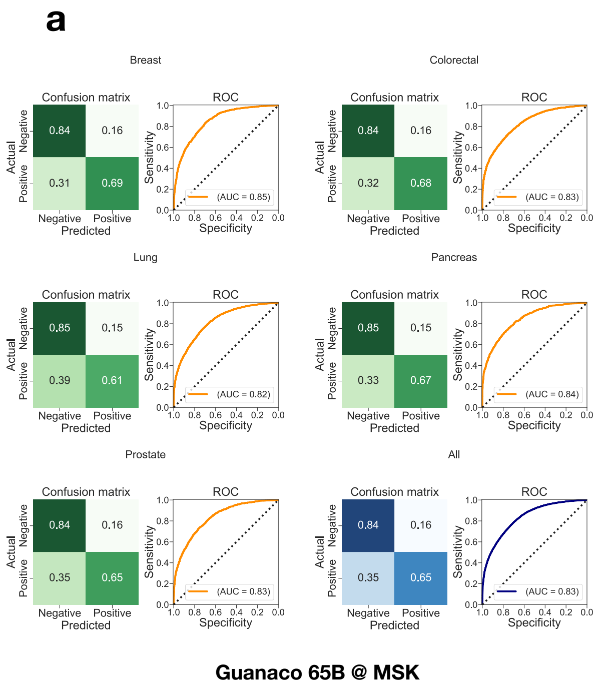

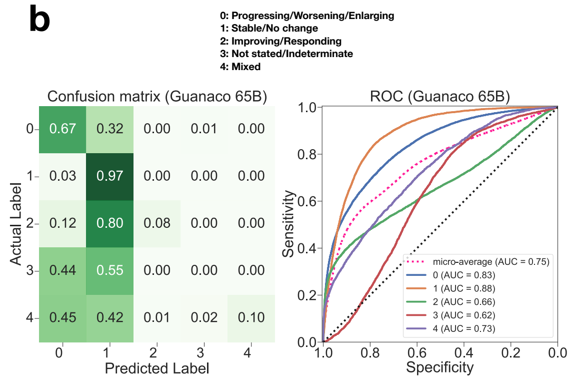

c **AUROC Comparison**

Model	Binary	Five Labels
**Woollie MSK 7B**	0.94	0.93
**Woollie 7B**	0.64	0.61
**Woollie MSK 33B**	0.97	0.97
**Wollie 33B**	0.89	0.84
**Guanaco 65B**	0.83	0.75

Supplementary Figure 7: Influence of prompt type on model performance.

The impact of prompt types on performance was analyzed using both Instruction and Chat prompts during the alignment process for the Woollie 7B, 13B, and 33B models and comparing their performance to benchmarks, including baseline Llama models for context. Chat prompts demonstrated superior performance across benchmarks in reasoning, conversation, and logic, particularly excelling in the medical domain. This highlights how prompt choice critically influences post-alignment model performance and is a crucial factor during the alignment phase.

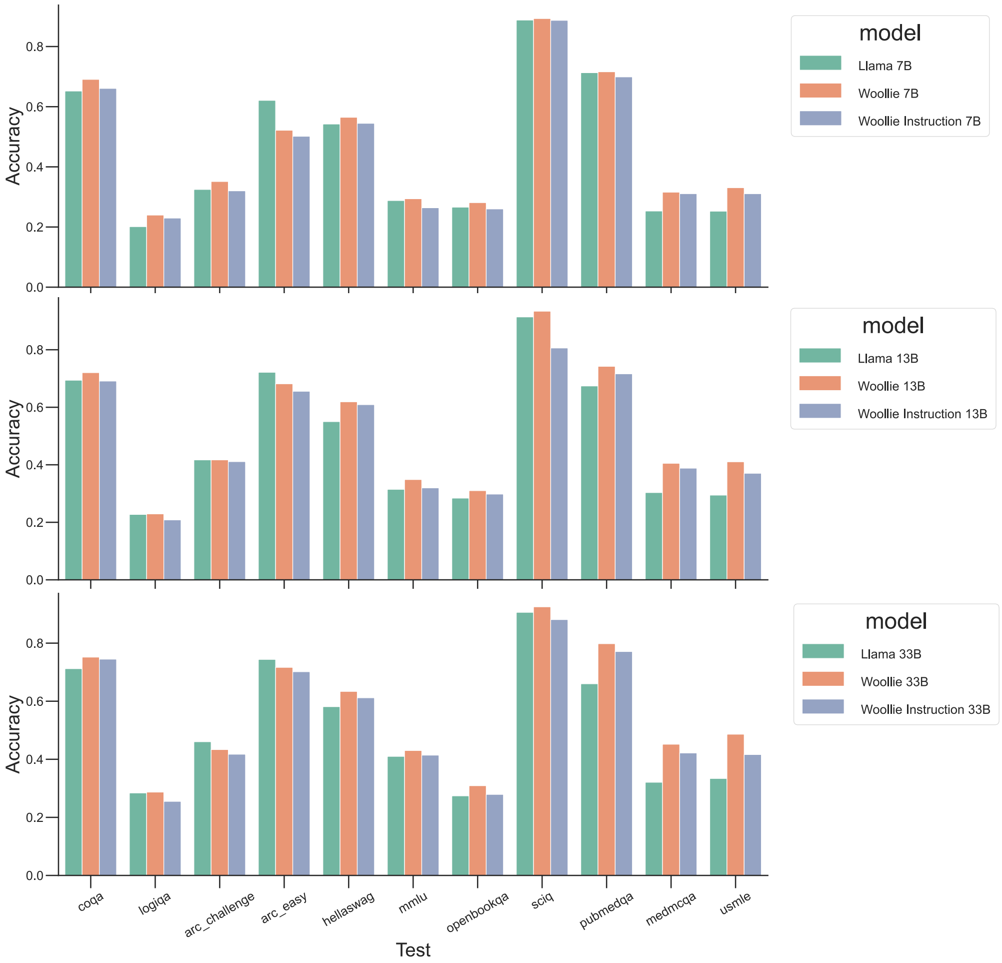
model
Llama 7B
Woollie 7B
Woollie Instruction 7B
model
Llama 13B
Woollie 13B
Woollie Instruction 13B
model
Llama 33B
Woollie 33B
Woollie Instruction 33B
Accuracy
0.0
0.2
0.4
0.6
0.8
coqa
logiqa
arc_challenge
arc_easy
hellaswag
mmlu
openbookqa
sciq
pubmedqa
medmcqa
usmle
Test

Supplementary Figure 8: The impact of few-shot learning on Woollie's performance

We experimented with 1-shot and 3-shot learning across Woollie models of different sizes. Due to the maximum token window of 2048 tokens, model performance dropped significantly beyond 3 shots. While 1-shot learning improved results across both non-medical and medical benchmarks, 3-shot learning did not yield substantial additional gains. This decline may be attributed to the model approaching its maximum context size, leading to a loss of focus on content in the middle of the sequence.

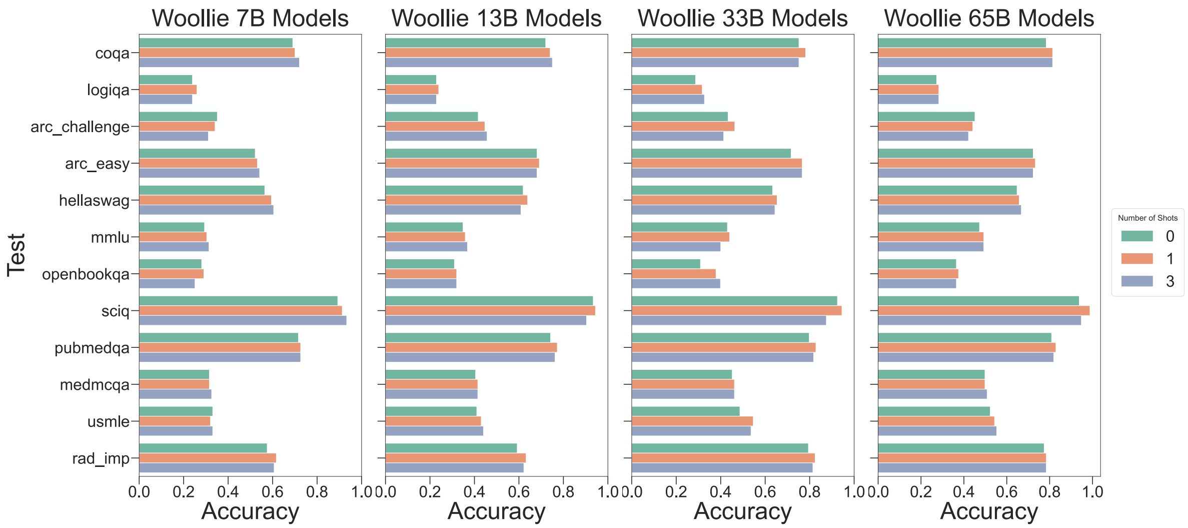
Woollie 7B Models
Woollie 13B Models
Woollie 33B Models
Woollie 65B Models
Test
coqa
logiqa
arc_challenge
arc_easy
hellaswag
mmlu
openbookqa
sciq
pubmedqa
medmcqa
usmle
rad_imp
Accuracy
0.0
0.2
0.4
0.6
0.8
1.0
Number of Shots
0
1
3

Supplementary Figure 9: Token distribution of the MSK radiology impression dataset

Box plots showing the token distribution in the MSK radiology impression dataset. Most notes contain fewer than 200 tokens, with a tail distribution extending to a maximum of 1250 tokens, well below the Woollie models' maximum token window of 2048 tokens. The dataset is categorized into five cancer types, with distributions shown for both negative-labeled notes ("Not Progressing") and positive-labeled notes ("Progressing"). All categories exhibit similar token distribution patterns.

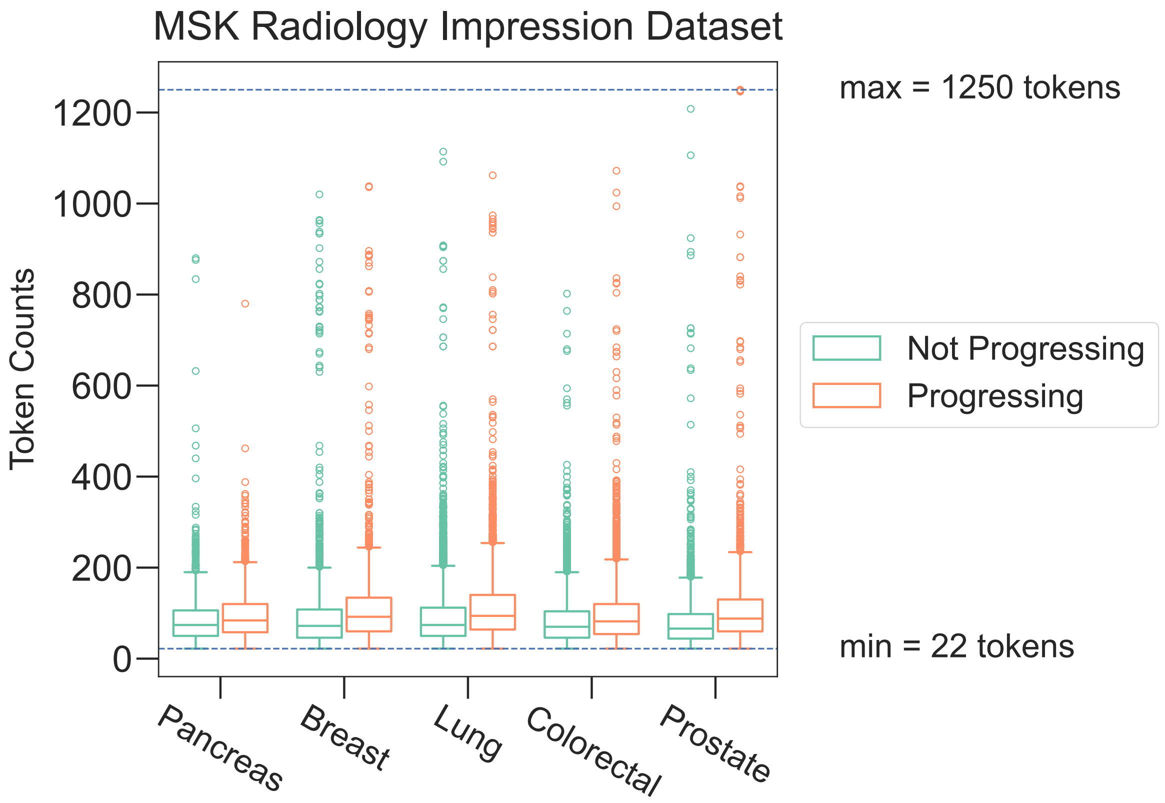

MSK Radiology Impression Dataset
max = 1250 tokens
min = 22 tokens
Token Counts
1200
1000
800
600
400
200
0
Not Progressing
Progressing
Pancreas
Breast
Lung
Colorectal
Prostate

Supplementary Figure 10: Woollie architecture diagram

Woollie models are aligned and fine-tuned on top of META's Llama (Llama 1) model, which is based on the transformer architecture. Llama is available in versions with 7B, 13B, 33B, and 65B parameters (actual parameter counts: 6.7B, 13B, 32.5B, and 65.2B, respectively). The number of heads in the multi-head attention mechanism for these variants are 32, 40, 52, and 64, respectively, compared to the original transformer, which had 8 heads. For the input embedding dimensions, the 7B model uses a 4096-dimensional vector, the 13B model uses 5120, the 33B model uses 6656, and the 65B model uses 8192 dimensions. In contrast, the original transformer architecture used 512-dimensional vectors to represent each token. Llama incorporates several innovations compared to the original transformer: RMS Normalization**:** Llama uses Root Mean Square (RMS) normalization instead of standard layer normalization. Rotary Positional Embedding**:** Llama employs rotary positional embeddings to dynamically learn positional representations. SwiGLU Activation Function**:** The feed-forward layer uses the SwiGLU activation function instead of the standard ReLU activation. These enhancements make Llama a more powerful and efficient architecture, forming the foundation for Woollie models.

Woollie (Llama) Architecture

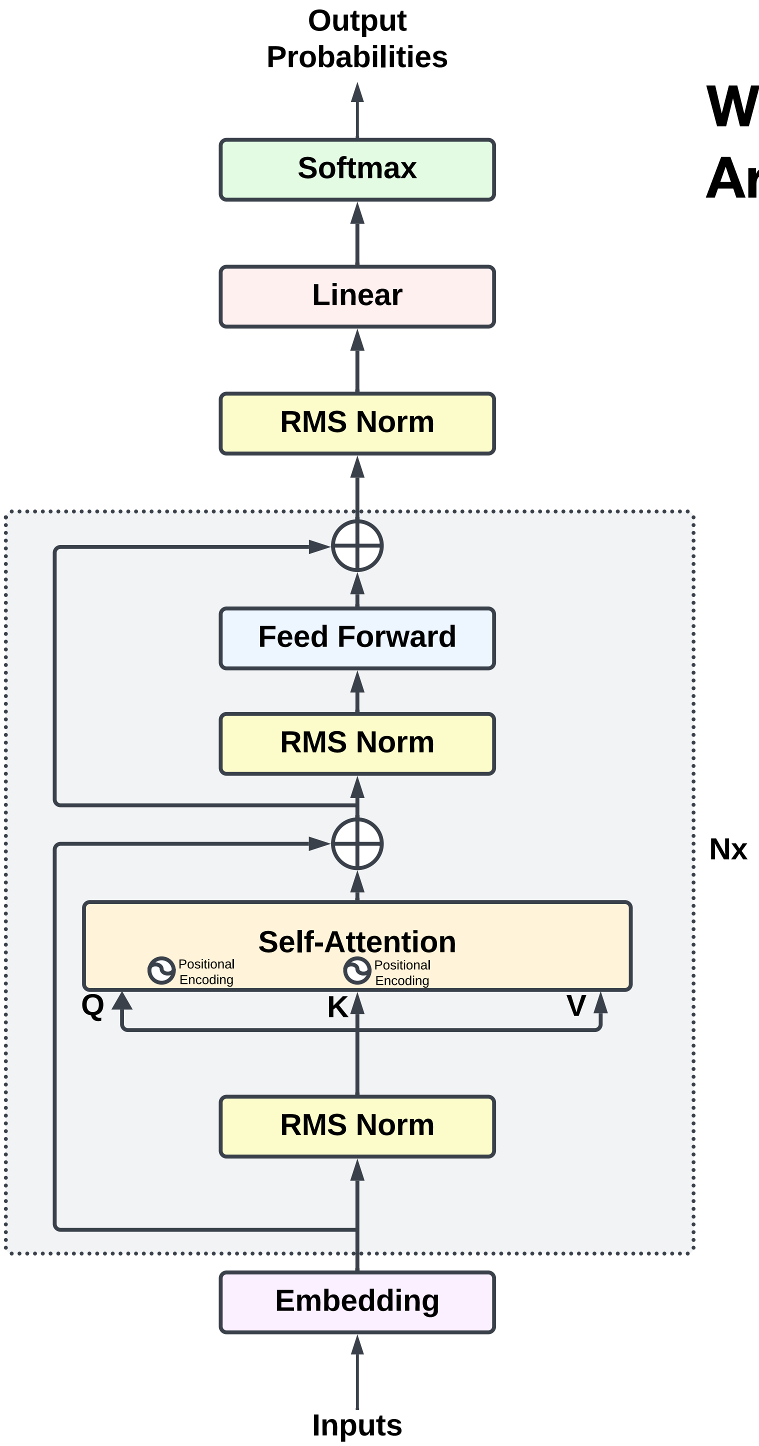

Supplementary Tables

Supplementary Table 1: List of metrics for models in this study

metric	coqa					logiqa					arc_challenge				
model	accuracy	precision	recall	f1	mcc	accuracy	precision	recall	f1	mcc	accuracy	precision	recall	f1	mcc
Llama 7B	0.65	0.70	0.70	0.68	0.18	0.20	0.23	0.23	0.20	0.12	0.32	0.39	0.38	0.38	0.18
Llama 13B	0.69	0.74	0.75	0.72	0.19	0.23	0.27	0.26	0.26	0.14	0.42	0.44	0.44	0.43	0.25
Llama 33B	0.71	0.76	0.78	0.74	0.21	0.28	0.29	0.29	0.28	0.15	0.46	0.48	0.48	0.47	0.30
Llama 65B	0.74	0.78	0.80	0.77	0.24	0.26	0.27	0.27	0.26	0.15	0.44	0.47	0.47	0.46	0.29
Woollie Foundation 7B	0.70	0.73	0.74	0.73	0.20	0.24	0.25	0.25	0.24	0.13	0.40	0.43	0.41	0.42	0.22
Woollie Foundation 13B	0.73	0.76	0.77	0.75	0.23	0.26	0.28	0.30	0.28	0.15	0.44	0.46	0.46	0.44	0.29
Woollie Foundation 33B	0.73	0.80	0.83	0.78	0.24	0.33	0.37	0.39	0.37	0.15	0.52	0.50	0.50	0.49	0.39
Woollie Foundation 65B	0.76	0.83	0.85	0.83	0.28	0.32	0.34	0.33	0.34	0.18	0.49	0.50	0.50	0.49	0.37
Woollie Medicine 7B	0.74	0.73	0.73	0.71	0.21	0.22	0.28	0.29	0.28	0.14	0.34	0.41	0.40	0.41	0.21
Woollie Medicine 13B	0.75	0.79	0.79	0.78	0.24	0.25	0.30	0.32	0.31	0.15	0.42	0.44	0.44	0.42	0.26
Woollie Medicine 33B	0.76	0.83	0.85	0.80	0.28	0.29	0.39	0.41	0.40	0.15	0.42	0.45	0.45	0.44	0.34
Woollie Medicine 65B	0.80	0.84	0.88	0.84	0.32	0.28	0.36	0.35	0.36	0.16	0.46	0.50	0.47	0.44	0.34
Woollie 7B	0.69	0.71	0.73	0.68	0.19	0.24	0.25	0.24	0.24	0.12	0.35	0.35	0.35	0.34	0.13
Woollie 13B	0.72	0.74	0.75	0.70	0.22	0.23	0.34	0.33	0.33	0.12	0.42	0.44	0.43	0.43	0.29
Woollie 33B	0.75	0.77	0.79	0.75	0.25	0.29	0.38	0.36	0.37	0.13	0.43	0.43	0.42	0.42	0.28
Woollie 65B	0.78	0.82	0.85	0.82	0.29	0.27	0.40	0.40	0.41	0.14	0.45	0.48	0.47	0.47	0.29
Woollie MSK 7B	0.68	0.70	0.71	0.66	0.18	0.22	0.30	0.29	0.29	0.16	0.40	0.41	0.41	0.40	0.23
Woollie MSK 33B	0.74	0.76	0.79	0.73	0.24	0.28	0.36	0.37	0.36	0.19	0.45	0.46	0.44	0.44	0.29

metric	arc_easy					hellaswag					mmlu				
model	accuracy	precision	recall	f1	mcc	accuracy	precision	recall	f1	mcc	accuracy	precision	recall	f1	mcc
Llama 7B	0.62	0.55	0.54	0.54	0.57	0.54	0.56	0.56	0.56	0.42	0.29	0.26	0.26	0.26	0.22
Llama 13B	0.72	0.59	0.59	0.59	0.65	0.55	0.59	0.59	0.59	0.45	0.31	0.29	0.31	0.29	0.47
Llama 33B	0.74	0.61	0.61	0.61	0.61	0.58	0.62	0.62	0.62	0.50	0.41	0.42	0.43	0.42	0.49
Llama 65B	0.73	0.61	0.60	0.60	0.68	0.59	0.64	0.64	0.64	0.52	0.44	0.50	0.50	0.50	0.62
Woollie Foundation 7B	0.69	0.62	0.61	0.61	0.60	0.57	0.59	0.59	0.59	0.48	0.31	0.28	0.28	0.28	0.26
Woollie Foundation 13B	0.77	0.64	0.63	0.63	0.69	0.61	0.59	0.59	0.59	0.45	0.34	0.32	0.32	0.31	0.49
Woollie Foundation 33B	0.80	0.65	0.64	0.64	0.67	0.64	0.66	0.66	0.66	0.56	0.44	0.45	0.44	0.45	0.52
Woollie Foundation 65B	0.77	0.65	0.63	0.63	0.71	0.65	0.70	0.70	0.70	0.61	0.48	0.55	0.55	0.55	0.70
Woollie Medicine 7B	0.62	0.59	0.58	0.58	0.57	0.55	0.59	0.59	0.59	0.51	0.29	0.29	0.29	0.29	0.30
Woollie Medicine 13B	0.70	0.61	0.61	0.61	0.61	0.60	0.56	0.56	0.56	0.40	0.33	0.33	0.31	0.30	0.45
Woollie Medicine 33B	0.75	0.61	0.60	0.59	0.60	0.62	0.65	0.65	0.65	0.52	0.42	0.42	0.42	0.42	0.48
Woollie Medicine 65B	0.75	0.66	0.61	0.60	0.68	0.64	0.69	0.69	0.69	0.61	0.46	0.56	0.56	0.56	0.68
Woollie 7B	0.52	0.41	0.41	0.41	0.55	0.56	0.55	0.55	0.55	0.41	0.29	0.28	0.28	0.28	0.23
Woollie 13B	0.68	0.60	0.59	0.59	0.60	0.62	0.58	0.58	0.58	0.44	0.35	0.32	0.31	0.31	0.49
Woollie 33B	0.72	0.61	0.51	0.51	0.52	0.63	0.62	0.62	0.62	0.49	0.43	0.44	0.46	0.43	0.53
Woollie 65B	0.72	0.61	0.61	0.60	0.58	0.65	0.62	0.62	0.62	0.49	0.47	0.54	0.54	0.54	0.59
Woollie MSK 7B	0.69	0.61	0.61	0.61	0.55	0.57	0.59	0.59	0.59	0.49	0.29	0.29	0.29	0.29	0.28
Woollie MSK 33B	0.79	0.59	0.58	0.59	0.62	0.64	0.65	0.65	0.65	0.59	0.43	0.44	0.43	0.43	0.51

metric	openbookqa					sciq					pubmedqa				
model	accuracy	precision	recall	f1	mcc	accuracy	precision	recall	f1	mcc	accuracy	precision	recall	f1	mcc
Llama 7B	0.27	0.29	0.28	0.28	0.05	0.89	0.75	0.73	0.74	0.70	0.71	0.50	0.55	0.52	0.53
Llama 13B	0.28	0.30	0.30	0.30	0.07	0.91	0.75	0.73	0.74	0.72	0.67	0.49	0.50	0.48	0.46
Llama 33B	0.27	0.31	0.31	0.31	0.08	0.91	0.78	0.77	0.76	0.76	0.66	0.51	0.50	0.47	0.47
Llama 65B	0.33	0.42	0.41	0.41	0.21	0.91	0.78	0.78	0.78	0.80	0.70	0.53	0.51	0.49	0.50
Woollie Foundation 7B	0.28	0.30	0.29	0.29	0.18	0.90	0.76	0.75	0.74	0.73	0.73	0.52	0.54	0.51	0.56
Woollie Foundation 13B	0.30	0.32	0.31	0.31	0.10	0.92	0.80	0.77	0.76	0.75	0.74	0.59	0.60	0.58	0.56
Woollie Foundation 33B	0.30	0.34	0.34	0.34	0.18	0.93	0.81	0.80	0.79	0.80	0.77	0.62	0.61	0.59	0.55
Woollie Foundation 65B	0.38	0.43	0.42	0.42	0.29	0.94	0.80	0.80	0.79	0.88	0.78	0.63	0.61	0.58	0.59
Woollie Medicine 7B	0.28	0.32	0.32	0.28	0.20	0.91	0.78	0.77	0.75	0.73	0.75	0.59	0.58	0.56	0.62
Woollie Medicine 13B	0.30	0.33	0.32	0.32	0.18	0.89	0.80	0.77	0.76	0.75	0.76	0.66	0.64	0.63	0.62
Woollie Medicine 33B	0.31	0.34	0.34	0.34	0.18	0.89	0.82	0.81	0.80	0.81	0.77	0.61	0.62	0.61	0.58
Woollie Medicine 65B	0.35	0.41	0.40	0.40	0.24	0.93	0.80	0.78	0.78	0.81	0.80	0.68	0.65	0.62	0.63
Woollie 7B	0.28	0.28	0.28	0.28	0.14	0.89	0.79	0.77	0.76	0.75	0.72	0.47	0.43	0.41	0.64
Woollie 13B	0.31	0.29	0.29	0.29	0.26	0.93	0.77	0.76	0.76	0.75	0.74	0.70	0.69	0.65	0.68
Woollie 33B	0.31	0.30	0.30	0.30	0.14	0.93	0.80	0.79	0.79	0.79	0.80	0.67	0.67	0.67	0.70
Woollie 65B	0.36	0.40	0.40	0.40	0.27	0.94	0.80	0.80	0.80	0.84	0.81	0.72	0.69	0.68	0.70
Woollie MSK 7B	0.28	0.30	0.30	0.30	0.14	0.88	0.77	0.75	0.73	0.69	0.79	0.59	0.58	0.57	0.68
Woollie MSK 33B	0.31	0.33	0.33	0.33	0.15	0.90	0.81	0.80	0.79	0.80	0.83	0.71	0.71	0.71	0.73

metric	medmcqa					usmle					rad_imp				
model	accuracy	precision	recall	f1	mcc	accuracy	precision	recall	f1	mcc	accuracy	precision	recall	f1	mcc
Llama 7B	0.25	0.28	0.27	0.22	0.03	0.25	0.26	0.26	0.24	0.02	0.49	0.46	0.90	0.61	0.08
Llama 13B	0.30	0.36	0.29	0.25	0.07	0.29	0.32	0.31	0.28	0.09	0.53	0.53	0.93	0.67	0.15
Llama 33B	0.32	0.40	0.34	0.32	0.14	0.33	0.45	0.39	0.37	0.20	0.60	0.53	0.97	0.69	0.35
Llama 65B	0.37	0.45	0.36	0.35	0.18	0.42	0.46	0.44	0.44	0.27	0.61	0.57	0.95	0.72	0.37
Woollie Foundation 7B	0.25	0.28	0.26	0.21	0.12	0.27	0.27	0.27	0.25	0.12	0.45	0.47	0.87	0.61	0.05
Woollie Foundation 13B	0.32	0.37	0.34	0.33	0.17	0.31	0.34	0.33	0.32	0.18	0.56	0.55	0.95	0.69	0.15
Woollie Foundation 33B	0.36	0.42	0.38	0.36	0.23	0.36	0.47	0.43	0.43	0.30	0.65	0.53	0.93	0.67	0.40
Woollie Foundation 65B	0.40	0.48	0.42	0.39	0.28	0.43	0.48	0.47	0.45	0.33	0.62	0.60	0.95	0.74	0.47
Woollie Medicine 7B	0.32	0.38	0.35	0.33	0.27	0.33	0.34	0.32	0.32	0.19	0.44	0.48	0.89	0.63	0.10
Woollie Medicine 13B	0.39	0.44	0.45	0.44	0.29	0.39	0.46	0.43	0.42	0.28	0.58	0.59	0.95	0.72	0.32
Woollie Medicine 33B	0.43	0.52	0.51	0.49	0.42	0.47	0.59	0.58	0.56	0.45	0.69	0.57	0.93	0.71	0.42
Woollie Medicine 65B	0.40	0.50	0.47	0.46	0.40	0.45	0.53	0.53	0.51	0.40	0.64	0.61	0.94	0.74	0.48
Woollie 7B	0.32	0.33	0.31	0.30	0.18	0.33	0.32	0.30	0.31	0.29	0.58	0.53	0.35	0.42	0.12
Woollie 13B	0.41	0.54	0.53	0.52	0.35	0.41	0.47	0.41	0.40	0.33	0.59	0.58	0.37	0.46	0.20
Woollie 33B	0.45	0.60	0.57	0.55	0.53	0.49	0.67	0.64	0.63	0.57	0.79	0.73	0.85	0.79	0.60
Woollie 65B	0.50	0.59	0.48	0.43	0.33	0.52	0.60	0.59	0.60	0.47	0.77	0.73	0.86	0.79	0.62
Woollie MSK 7B	0.35	0.38	0.38	0.36	0.30	0.38	0.42	0.40	0.41	0.39	0.86	0.93	0.75	0.83	0.73
Woollie MSK 33B	0.48	0.65	0.62	0.60	0.59	0.53	0.73	0.69	0.68	0.69	0.90	0.86	0.92	0.90	0.80

Supplementary Table 2: Model resource consumption and inference time

Model size	GPU memory allocation (%)	GPU power consumption (W)	GPU utilization (%)	Processor temperature (℃)	Token generation (token/s)
7B	20	180	60	60	6
13B	34	202	68	64	4
33B	84	225	71	63	2
65B (4bit)	95	267	82	65	0.7

Supplementary Table 3: Alignment and fine-tune hyper-parameters for all Woollie models

Model size	r	a	Dropout	Micro batch size	Global batch size	Gradient accumulation	Learning rate
7B	8	16	0.05	8	128	2	3×10^{-4}
13B	8	16	0.05	8	128	2	3×10^{-4}
33B	8	16	0.05	2	128	4	3×10^{-4}
65B	64	16	0.05	1	64	2	1×10^{-4}

Supplementary Table 4: Selection of benchmarks utilized for evaluating our alignment and fine-tuning approaches

Tests	Description	Conversation	Q & A	Topics	Year	Size
**COQA**	Conversational Question Answering	Yes	No	• Children's Stories<br>• Literature<br>• Mid/High School Exams<br>• News<br>• Wikipedia<br>• Reddit<br>• Science	2019	8k
**LogiQA**	A Challenge Dataset for Machine Reading Comprehension with Logical Reasoning	No	Yes	• Logical reasoning<br>• Civil Servants Exams	2020	8k
**ARC easy**	AI2 Reasoning Challenge (ARC)	No	Yes	• Grade-school natural science	2018	5k
**ARC hard**	AI2 Reasoning Challenge (ARC)	No	Yes	• From ARC easy dataset, those that both a retrieval and a co-occurrence method fail to answer correctly	2018	2k
**HellaSWAG**	Sentence completion	Yes	No	• WikiHow	2018	40k
**MMLU**	Massive Multitask Language Understanding	No	Yes	• 57 tasks including:<br>○elementary mathematics<br>○U S history<br>○Computer science<br>○Law<br>○Anatomy<br>○etc.	2020	116k
**OpenBookQA**	Open Book Question Answering it contains questions that require multi-step reasoning, use of additional common and commonsense knowledge, and rich text comprehension.	No	Yes	• Elementary level questions with facts as context	2018	10k
**SciQ**	Crowdsourcing Multiple Choice Science Questions	No	Yes	• Physics<br>• Chemistry<br>• Earth Science<br>• Biology<br>• Anatomy	2017	12k
**PubMedQA**	Dataset for Biomedical Research Question Answering	No	Yes	• Biomedical researches from NIH PubMed (https://pubmed.ncbi.nlm.nih.gov/)	2019	211k
**USMLE**	United States Medical License Exams (USMLE). The dataset is collected from the professional medical board exams.	No	Yes	• USMEL Step-1<br>• USMLE Step-2 CK	2022	10k
**MedMCQA**	MedMCQA is a large-scale, Multiple-Choice Question Answering (MCQA) dataset designed to address real-world medical entrance exam questions.	No	Yes	• Anesthesia<br>• Anatomy<br>• Biochemistry<br>• Dental<br>• ENT<br>• Forensic Medicine (FM)<br>• Obstetrics and Gynecology (O&G)<br>• Medicine<br>• Microbiology<br>• Ophthalmology<br>• Orthopedics<br>• Pathology<br>• Pediatrics<br>• Pharmacology<br>• Physiology<br>• Psychiatry<br>• Radiology<br>• Skin<br>• Preventive & Social Medicine (PSM)<br>• Surgery	2022	183k

Supplementary Table 5: Comparison of model performance across sociodemographic groups and cancer types.

a), Model performances were observed across “Age”, “Birth sex”, and “Race”. b), Model performance varied across different cancer types.

a

	Woollie MSK 7B @MSK						Woollie MSK 33B @MSK						Woollie MSK 7B @UCSF						Woollie MSK 33B @UCSF					
	accuracy	precision	recall	f1	mcc	auroc	accuracy	precision	recall	f1	mcc	auroc	accuracy	precision	recall	f1	mcc	auroc	accuracy	precision	recall	f1	mcc	auroc
**Age**																								
Under 40s	0.88	0.94	0.77	0.85	0.75	0.94	0.89	0.87	0.90	0.88	0.76	0.96	0.75	0.76	0.98	0.86	0.60	0.91	0.85	0.98	0.90	0.94	0.77	0.94
41-50	0.86	0.93	0.70	0.80	0.73	0.93	0.90	0.87	0.92	0.89	0.79	0.96	0.85	0.82	0.99	0.90	0.63	0.90	0.90	0.99	0.85	0.91	0.79	0.88
51-60	0.87	0.94	0.73	0.82	0.72	0.92	0.88	0.82	0.89	0.85	0.76	0.97	0.75	0.77	0.93	0.84	0.51	0.84	0.80	0.88	0.75	0.81	0.70	0.95
61-70	0.88	0.93	0.72	0.81	0.71	0.94	0.91	0.89	0.90	0.89	0.82	0.97	0.75	0.78	0.91	0.84	0.55	0.94	0.80	0.97	0.74	0.84	0.68	0.92
71-80	0.85	0.94	0.73	0.82	0.72	0.94	0.91	0.89	0.89	0.89	0.82	0.98	0.84	0.71	0.98	0.82	0.50	0.89	0.80	0.85	0.75	0.80	0.67	0.81
81+	0.83	0.92	0.69	0.79	0.69	0.93	0.88	0.83	0.87	0.85	0.72	0.96	0.85	0.87	0.92	0.89	0.59	0.92	0.75	0.95	0.80	0.87	0.69	0.97
p-value	***0.12***	***0.51***	***0.16***	***0.15***	***0.15***	***0.80***	***0.14***	***0.13***	***0.73***	***0.12***	***0.14***	***0.50***	***0.16***	***0.23***	***0.70***	***0.28***	***0.13***	***0.82***	***0.62***	***0.13***	***0.41***	***0.45***	***0.69***	***0.94***
**Birth Sex**																								
Male	0.85	0.93	0.74	0.82	0.73	0.94	0.89	0.86	0.93	0.89	0.78	0.96	0.79	0.71	0.91	0.80	0.57	0.88	0.85	0.95	0.88	0.91	0.66	0.83
Female	0.86	0.94	0.77	0.85	0.74	0.92	0.89	0.85	0.93	0.89	0.78	0.95	0.85	0.82	0.98	0.89	0.60	0.98	0.89	0.92	0.82	0.87	0.73	0.98
p-value	***0.71***	***0.90***	***0.72***	***0.81***	***0.79***	***0.20***	***0.82***	***0.81***	***0.98***	***0.98***	***0.95***	***0.51***	***0.54***	***0.55***	***0.42***	***0.51***	***0.95***	***0.42***	***0.69***	***0.89***	***0.59***	***0.61***	***0.30***	***0.42***
**Race**																								
White	0.86	0.94	0.76	0.84	0.73	0.94	0.88	0.85	0.92	0.88	0.76	0.95	0.78	0.83	0.89	0.86	0.60	0.87	0.70	0.98	0.65	0.78	0.67	0.95
African American	0.83	0.95	0.73	0.83	0.71	0.94	0.90	0.91	0.91	0.91	0.81	0.95	0.85	0.82	0.88	0.85	0.65	0.94	0.90	0.90	0.85	0.87	0.78	0.94
Asian/Indian	0.87	0.92	0.75	0.83	0.73	0.96	0.90	0.83	0.95	0.89	0.80	0.98	0.80	0.78	0.92	0.84	0.50	0.9	0.80	0.89	0.70	0.78	0.66	0.87
No Answer	0.89	0.95	0.78	0.86	0.78	0.94	0.90	0.88	0.90	0.89	0.80	0.98	-	-	-		-		-	-	-	-	-	-
Other	0.90	0.97	0.79	0.87	0.80	0.97	0.90	0.82	0.95	0.88	0.80	0.98	0.80	0.81	0.98	0.89	0.63	0.83	0.98	0.95	0.95	0.95	0.95	0.98
p-value	***0.15***	***0.44***	***0.76***	***0.68***	***0.23***	***0.29***	***0.84***	***0.28***	***0.21***	***0.76***	***0.89***	***0.27***	***0.12***	***0.14***	***0.78***	***0.13***	***0.16***	***0.48***	***0.29***	***0.35***	***0.13***	***0.20***	***0.20***	***0.51***

b

	Woollie MSK 7B @MSK						Woollie MSK 33B @MSK						Woollie MSK 7B @UCSF						Woollie MSK 33B @UCSF					
	accuracy	precision	recall	f1	mcc	auroc	accuracy	precision	recall	f1	mcc	auroc	accuracy	precision	recall	f1	mcc	auroc	accuracy	precision	recall	f1	mcc	auroc
Breast	0.85	0.94	0.76	0.85	0.73	0.94	0.88	0.86	0.93	0.89	0.76	0.96	0.79	0.80	0.95	0.87	0.68	0.87	0.69	0.94	0.62	0.75	0.63	0.86
Colorectal	0.88	0.94	0.81	0.87	0.77	0.96	0.91	0.88	0.93	0.90	0.82	0.97	-	-	-	-	-	-	-	-	-	-	-	-
Lung	0.86	0.91	0.71	0.80	0.71	0.93	0.89	0.84	0.89	0.87	0.78	0.96	0.88	0.88	0.95	0.91	0.72	0.94	0.78	0.99	0.67	0.80	0.73	0.95
Pancreas	0.88	0.97	0.74	0.84	0.76	0.96	0.92	0.90	0.93	0.91	0.84	0.98	-	-	-	-	-	-	-	-	-	-	-	-
Prostate	0.84	0.91	0.71	0.80	0.69	0.92	0.89	0.86	0.91	0.89	0.79	0.96	0.72	0.66	0.92	0.77	0.67	0.87	0.67	0.90	0.40	0.55	0.62	0.81
p-value	***< 0.01***						***< 0.01***						***< 0.01***						***< 0.01***					
